# Salvia miltiorrhiza in osteoporosis: a review of its phytochemistry, traditional clinical uses and preclinical studies (2014–2024)

**DOI:** 10.3389/fphar.2024.1483431

**Published:** 2024-10-03

**Authors:** Lili Wang, Shan Wang, Xuan Dai, Gaiyue Yue, Jiyuan Yin, Tianshu Xu, Hanfen Shi, Tianyuan Liu, Zhanhong Jia, Dieter Brömme, Shuofeng Zhang, Dongwei Zhang

**Affiliations:** ^1^ Department of TCM Pharmacology, Chinese Material Medica School, Beijing University of Chinese Medicine, Beijing, China; ^2^ Diabetes Research Center, School of Traditional Chinese Medicine, Beijing University of Chinese Medicine, Beijing, China; ^3^ Department of Oral Biological and Medical Sciences, Faculty of Dentistry, The University of British Columbia, Vancouver, Canada

**Keywords:** osteoporosis, *Salvia Miltiorrhiza*, preclinical investigations, TCM clinical trials, Osteoblast, Osteoclast

## Abstract

Osteoporosis becomes a global public health concern due to its rising prevalence and substantial impact on life quality. Salvia miltiorrhiza Bunge (Salviae Miltiorrhizae Radix et Rhizoma, SM) has been firstly recorded in Shen Nong’s Herbal Classic, and is frequently prescribed in conjunction with other herbs for the management of osteoporosis. This systematic review aims to comprehensively analyze the recent advances of SM on osteoporosis in traditional Chinese clinical uses and preclinical investigations. Literature encompassing pertinent studies were systematically retrieved across multiple databases, including the PubMed, Web of Science, Chinese National Knowledge Infrastructure, Chinese VIP Database, and Chinese Biomedical Literature Database. Original investigations spanning from February 2014 to March 2024, including traditional Chinese medicine (TCM) clinical trials and preclinical studies, were employed to analyze the effects and actions of SM on osteoporosis. Thirty-eight TCM clinical trials were identified to employ SM in combination with other herbs for the management of primary and secondary osteoporosis. The overall efficacy was between 77% and 96.67%. Forty preclinical studies were identified to investigate the effects and actions of SM and/or its ingredients on osteoporosis. The anti-osteoporosis actions of this herb may be attributed to inhibit osteoclastogenesis/bone resorption and promote osteoblastogenesis/osteogenesis. The ethanol extracts and its ingredients (tanshinones) inhibit osteoclastogenesis/bone resorption by inhibiting the MAPK/NF-κB/NFATc1 signaling pathway and cathepsin K-induced collagen degradation. Both ethanol extracts (tanshinones) and water extracts (Sal B and tanshinol) contribute to osteoblastogenesis by promoting osteogenesis and angiogenesis via activation of the Wnt/β-catenin/VEGF and ERK/TAZ pathways, and eliminating ROS production targeting Nrf2/ARE/HO-1 pathway. In conclusions, SM may offer a novel strategy for osteoporosis management. Well-designed clinical trials are still needed to evaluate the actions of this herb and its ingredients on bone remodeling.

## 1 Introduction

Osteoporosis is one type of degenerative bone diseases characterized by diminished bone density and disorganized bone microarchitecture, resulting in compromised bone strength and higher risk of fracture (Ensrud and Crandall, 2024). Osteoporosis attacks both genders, yet exhibits a higher prevalence among women aged 50 or older compared to their male counterparts. This sex disparity primarily attributed to the abrupt cessation of estrogen, precipitating heightened bone turnover rates and subsequently accelerating bone loss. According to the recent epidemiology, approximately 50% of women and 20% of men aged 50 or older in the USA are projected to experience osteoporotic fracture during their remaining lifetime. By 2040, the global incidence of osteoporosis aged 50 or older is estimated to be doubled from 158 million individuals in 2010 (Oden et al., 2015). By 2025, the cost of osteoporosis-related fractures in USA are projected to increase to US$253 billion per year (Burge et al., 2007). This places a huge economic burden on the healthcare system of the society.

Current FDA-approved drugs for osteoporosis can be divided into anti-resorptive inhibitors and osteoanabolic medications, including anti-resorptive bisphosphonates (Alendronate, Zoledronic acid), estrogen receptors modulators, estrogen replacement, receptor activator of nuclear factor kappa-Β ligand (RANKL) inhibitors (Denosumab) and osteoanabolic parathyroid hormone analogs (Teriparatide, Abaloparatide), sclerostin inhibitor (Romosozumab) (LeBoff et al., 2022). These drugs are documented to effectively prevent bone loss but exhibit obvious side effects (Lim, 2022; Handel et al., 2023; Ayers et al., 2023; Kendler et al., 2022; Kostenuik et al., 2023). For example, anti-resorptive drugs may inhibit bone formation when bone resorption is limited (Ekeuku et al., 2021), owing to the coupling between osteoblasts and osteoclasts (Ekeuku et al., 2021). Due to the chronic characters of this disease, years or even decades of interventions may be needed. However, long-term use of anti-resorptive bisphosphonates and denosumab may increase risk of atypical femoral fractures and osteonecrosis of the jaw (Ayers et al., 2023; Lim, 2022). Hence, there is an imperative to develop novel count-measures with low or no side effects for long-term management of osteoporosis.

**FIGURE 1:**
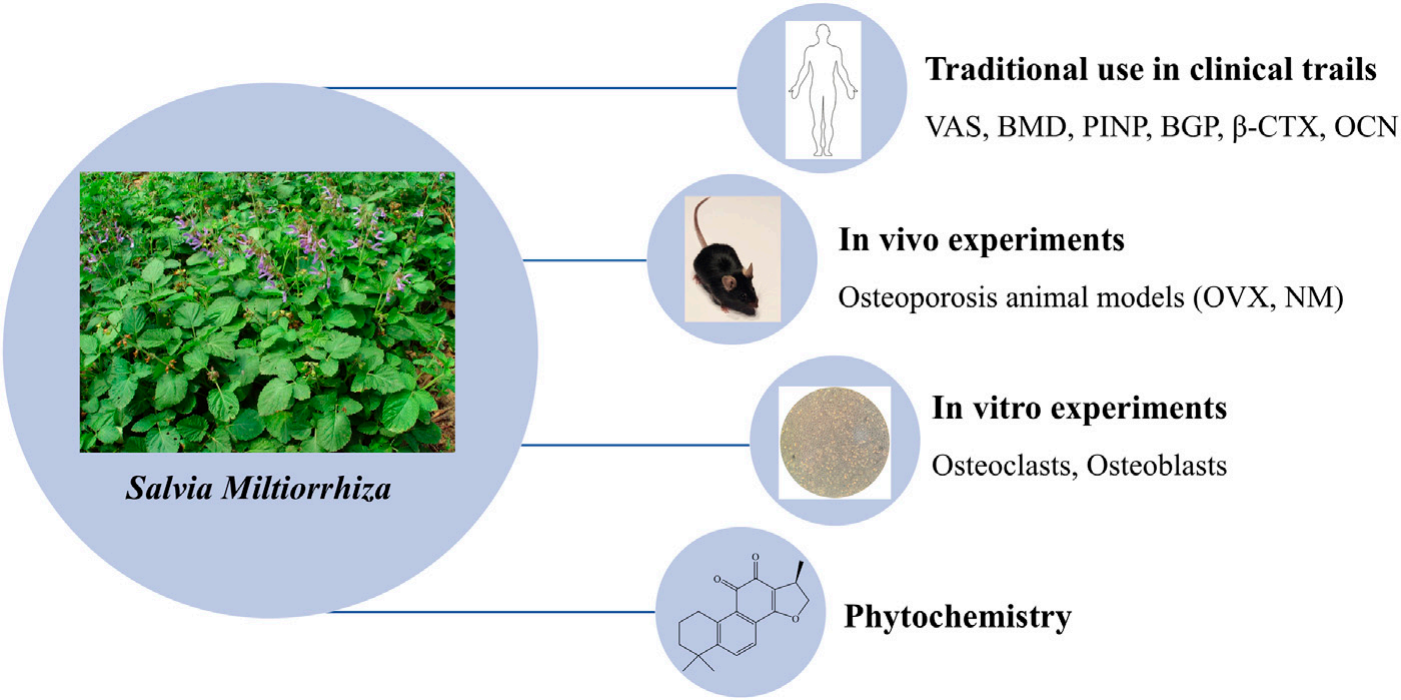
A sketch of SM in osteoporosis on its Phytochemistry, Traditional Clinical Uses and Preclinical Studies.

The dried root of *Salvia miltiorrhiza* Bunge (Salviae Miltiorrhizae Radix et Rhizoma, SM), also named as “Danshen,” “Red sage,” was used as a traditional and folk Chinese medicine in many Asian countries, especially in China and Japan, with markets in America and Europe growing substantially (Kum et al., 2021). It has been historically and is currently either used alone or in combination with other herbs to treat skeletal diseases (Guo et al., 2014). In the previous investigation, we have reviewed the applications of SM in the management of osteoporosis *in vivo* and *in vitro* from 2003 to 2013, revealing the potential of SM as a novel source of anti-osteoporotic agents (Guo et al., 2014). Notably, after searching and researching for another decade, there is emerging evidence to support this potential. Therefore, we further analyze the recent progress in SM for the treatment of osteoporosis from 2014 to 2024. For this purpose, we will firstly summarize the clinical uses of SM to treat patients with osteoporosis in traditional Chinese medicine (TCM) trials. And then, we examine the recent advance of the phytomedicine of this herb. Lastly, we comprehensively review the actions and underlying mechanisms of SM on bone remodeling, including evidence derived from both *in vitro* and *in vivo* studies (Figure 1).

## 2 Traditional Definition of *Salvia Miltiorrhiza* on osteoporosis

SM was highly valued and recorded in some Chinese herbal classics such as Shen Nong’s Herbal Classic of the Materia Medica (Shennong Bencao Jing), Compendium of Materia Medica (Bencao Gangmu) and Chinese Materia Medica (Zhonghua Bencao) (Su et al., 2015) and is noted for its efficacy in promoting blood circulation and resolving blood stasis, relieving pain and swelling (Kum et al., 2021).

According to the TCM theories “kidney governing bone,” “spleen dominating muscles,” “liver storing blood,” “Qi and blood nourishing bone,” the main pathogenesis of osteoporosis is attributed to deficiencies in kidney, liver and spleen, which are linked to “a “stagnation” and “lack of flow” of blood.” Thusly, the occurrence of blood stasis and “lack of flow” contributes to the bone loss, muscle lose, joint failure and bone pain (Wu et al., 2024; Liu et al., 2024a). Therefore, promoting blood circulation to remove blood stasis is a strategy to treat osteoporosis. This highlights the potential of SM to treat osteoporosis.

## 3 Methodology

**FIGURE 2:**
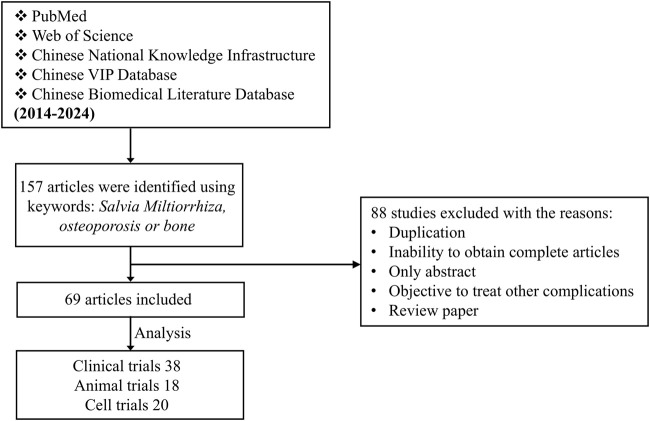
Flow chart of literature analysis.

To fully understand the actions of SM on osteoporosis, the following databases were used for retrieving the information: PubMed (www.pubmed.com), CNKI (www.cnki.net), the Web of Science (www.webofscience.com), www.cqvip.com, and www.wanfangdata.com (Figure 2). The following words and/or phrases in various combinations were employed to retrieve the relevant references: *S. Miltiorrhiza*, osteoporosis, bone, osteoblasts, osteoclasts, clinical trials. Publications were initially screened by title and abstract.

Approximately 157 papers were retrieved from January 2014 to June 2024. Of these, 88 papers were excluded with the following criteria: duplication, only abstract, reviews and objective to treat other complications (Figure 2).

## 4 *Salvia Miltiorrhiza* in TCM clinical trials of osteoporosis

Around 36 TCM clinical observations appeared during the past decades. Liu et al. (2014) evaluated 16 trials investigating the anti-osteoporotic efficacy with distinct Chinese herbal formulations including SM. And then Guo et al. (2014) evaluated 20 trials investigating the efficacy of SM in combination with other herbal and non-botanical components such as extracts from ants, shells, Chinese pangolin, and fossil bones, for the treatment of primary and secondary osteoporosis. The combined 36 trials totally enrolled 2066 patients, from which 25 trails targeted primary osteoporosis (including 13 trials of post-menopausal osteoporosis, 2 trials of senile osteoporosis), 8 trials treated secondary osteoporosis (major diabetes-induced osteoporosis), and 3 trials did not state explicitly the osteoporosis type.

In addition to the 36 TCM trails reported by Liu et al. (2014) and Guo et al. (2014), we have identified an additional 38 clinical studies focusing on primary and secondary osteoporosis that employed 33 different Chinese herbal prescriptions including SM in combination with other botanicals and non-botanical components. Among these, only 3 studies used single SM or its ingredients (Tanshinone IIA) to treat osteoporotic patients (Supplementary Table S1). These 38 clinical trials included 1,611 patients in total. Among which, 28 trials targeted primary osteoporosis, including post-menopausal, perimenopausal, and senile osteoporosis. For the rest, 6 trials focused on the treatment of patients with diabetes-induced osteoporosis, 4 trials did not indicate the type of osteoporosis (Supplementary Table S1). In addition, among the clinical observations, 35 investigations are single center randomized controlled trial (RCT), while only 1 investigation is double-blind multicenter randomized placebo-controlled trial. And 2 investigations did not give the types of clinical trials.

These trials determined anti-osteoporosis effects of this herb by testing bone mineral density (BMD), bone metabolism markers, as well as clinical symptoms such as osteoporotic pain, and life quality (Supplementary Table S1). Bone metabolism markers included degradation products of bone collagen molecules (C-telopeptide cross-linked type 1 collagen [CTX-1]), enzymatic activities reflecting osteoclastic resorption, tartrate-resistant acid phosphatase [TRACP or TRAP]), type 1 collagen synthesis (procollagen type I N-propeptide [PINP]), osteoblast enzymes (bone-specific alkaline phosphatase [BALP]), or bone matrix proteins (osteocalcin [OCN] and bone Gla protein [BGP]) (Brown et al., 2022). High efficacy was predominantly characterized by a rise in BMD > 0.02 g/cm^2^ coupled with alleviation of osteoporotic pain and improvement of bone metabolism markers (serum CTX-1, PINP, TRACP, Ca, P, and OCN). The collective efficacy (with marked and moderate improvements) of trials involving patients with primary osteoporosis ranged from 77% to 96.67%. And that for patients with secondary osteoporosis, the efficacy ranged from 83.3% to 93.3% (Supplementary Table S1).

It should be noted that in the control groups the most frequently used western medicine were vitamin D and calcium supplements (Caltrate D, Calcium carbonate D3 calcitriol), bone resorption inhibitors calcitonin (Salmon calcitonin) and bisphosphonates (Alendronate sodium, Zoledronic acid). While the novel medicine including RANKL inhibitor (Denosumab), and selective estrogen receptor modulators (Raloxifene), as well as osteoanabolic parathyroid hormone analogs (Teriparatide, Abaloparatide), were not included. The overall efficacy of trails used Chinese herbal prescriptions including SM or integrated these prescriptions with western medicine were better than those of western medicine only. (Supplementary Table S1).

Most of the studies (20 trials) reported absences of adverse reactions during the therapeutic interventions. Eight trials did not report side effects. Ten trails reported slight adverse reactions, among which 1 patient had pain and red skin in infusion site caused by quick infusion (Yang et al., 2017), 15 cases had slight gastrointestinal reactions such as nausea, vomiting, constipation, diarrhea (Chen et al., 2020; Yang, 2015; Dai, 2022; Han, 2016; Hong, 2022; Wang, 2022), 2 cases had dizziness (Wang, 2022; Su et al., 2020), 4 cases had skin rash (Chen et al., 2020; Wang, 2022; Su et al., 2020), 1 case had nausea, vomiting and dizziness (Su et al., 2020), 1 case decreased white blood cells and 1 case had abnormal liver function (Shuai, 2018). However, the similar or even higher incidence of side effects were observed in the control group (western medicine only or western medicine plus Chinese medicine) (Supplementary Table S1).

Although these TCM clinical trials have demonstrated that SM were effective and safe for the treatment of osteoporosis, most of these clinical trials are not well designed. Owing to the chronic characters of osteoporosis, years or even decades of preventive measures or therapy are needed (Foessl et al., 2023). However, all the 38 clinical trials are characterized by a rather short treatment duration from 2 weeks to 6 months. In addition, all the 38 clinical trials included a very small number of patients from 5 to 100 cases and were not placebo controlled. These make the evidence of efficacy of this herb is discounted. Besides, reducing fracture rate is one of the main aims of osteoporosis control (Kelm et al., 2023), while none of the 38 clinical trials addressed the effects of these TCM prescriptions or single SM products on it. Furthermore, the outcomes for the trials mostly included ambiguously description with the evaluated parameters and biomarkers, thus making it difficult to overall compare and validate the studies. Therefore, large randomized clinical trials with defined and unified criteria for clinical efficacy will be necessary to better understanding of its action in the future clinical studies.

In these TCM clinical trials, numerous preparations of SM products or TCM prescriptions are used, including tablets/pill, capsules, granules, injectables, oral liquids (soup/decoction). These TCM prescriptions used in the osteoporotic trials mostly contained 6 ∼ 18 individual herbs or non-plant components. The relative weight contributions of SM in these formulations ranged from 5% to 30%, as detailed in Supplementary Table S1. However, these available data failed to establish a discernible correlation between the relative weight of SM and the efficacy observed in the trials. Owing to the substantial variability in herbal compositions (number of herbs used and their respective quantities), it is difficult to comprehensively evaluate the efficacy of this herb on osteoporosis.

Li et al. (2022) collected 369 Chinese medicine prescriptions and found that the top 3 frequently used herbs in the treatment of postmenopausal osteoporosis were *Epimedium brevicornu* Maxim. (Epimedii Folium), *Rehmannia glutinosa* Libosch. (Rehmanniae Radix Praeparata) and *Angelica sinensis* (Oliv.) Diels (Angelicae Sinensis Radix). Although SM did not emerge as top herbs among the most used ones in TCM osteoporosis clinical trials, a thorough search on PubMed using the keywords “*S. Miltiorrhiza*” in conjunction with “bone” yielded 123 pertinent articles. Other 10 most frequently utilized medicinal herb in the 38 studies that included SM are compiled in Table 1.

**TABLE 1:** Most frequently herbs used in combination with *Salvia Miltiorrhiza* in osteoporosis clinical trials.

Name of herb (Chinese pinyin name)	Frequency
Epimedii Folium (Yinyanghuo)	23
Rehmanniae Radix Praeparata (Shudi)/Rehmanniae Radix (Shengdi)	20
Achyranthis Bidentatae Radix (Niuxi)	19
Astragali Radix (Huangqi)	18
Dioscoreae Rhizoma (Shanyao)	17
Drynariae Rhizoma (Gusuibu)	16
Angelicae Sinensis Radix (Danggui)	14
Eucommiae Cortex (Duzhong)	14
Glycyrrhizae Radix ET Rhizoma (Gancao)	12
Corni Fructus (Shanzhuyu)	12

## 5 Phytochemistry of *Salvia Miltiorrhiza*

Currently, more than 100 compounds were characterized from *Salvia miltiorrhiza* Bunge, which can be divided into two major groups: water-soluble (hydrophilic) compounds and lipid-soluble (lipophilic) compounds. The water-soluble/hydrophilic compounds are mainly phenolic acids including salvianolic acid A/B/C, and rosmarinic acid. Lipid-soluble/lipophilic compounds are various nonpolar diterpenoid quinones including cryptotanshinone (CPT), tanshinone I/IIA/IIB/V/VI, dihydrotanshinone І (DHT), isotanshinone I/II (Guo et al., 2014; Jia et al., 2019; Lin and Chang, 2000; Zhao et al., 2022).

Recent years, more and more diterpenoid quinones have been identified from this herb. Yin et al. (2021) isolated 13 novel diterpenoid quinones from the dried roots of SM, including R-(+)-salmiltiorin E, R-(+)-grandifolia D, salvianone ester A, which is reported to exhibit anti-tumor activities. Ying-Jie Ren and his team further isolated 25 novel diterpenoid quinones, including salviamilthone A/B/C/D/E/F/G/H/I/J/K/L/M/N/O, salvianolactone acid I, epi-danshenspiroketallactone F, danshinspiroketallactone, grandifolia G, which is demonstrated to exihibit anti-inflammatory effects (Ren et al., 2023; Ren et al., 2022). In addition, Jiang et al. (2023) isolated 6 tanshinones, including (+)-2-Cl-tanshindiol C, (−)-2-Cl-tanshindiol C, (+)-tanshinoic acid D, (−)-tanshinoic acid D, (−)-tanshinoic acid E, and (+)-tanshinoic acid E. All of these compounds were documented to exhibit antioxidant activities.

**FIGURE 3:**
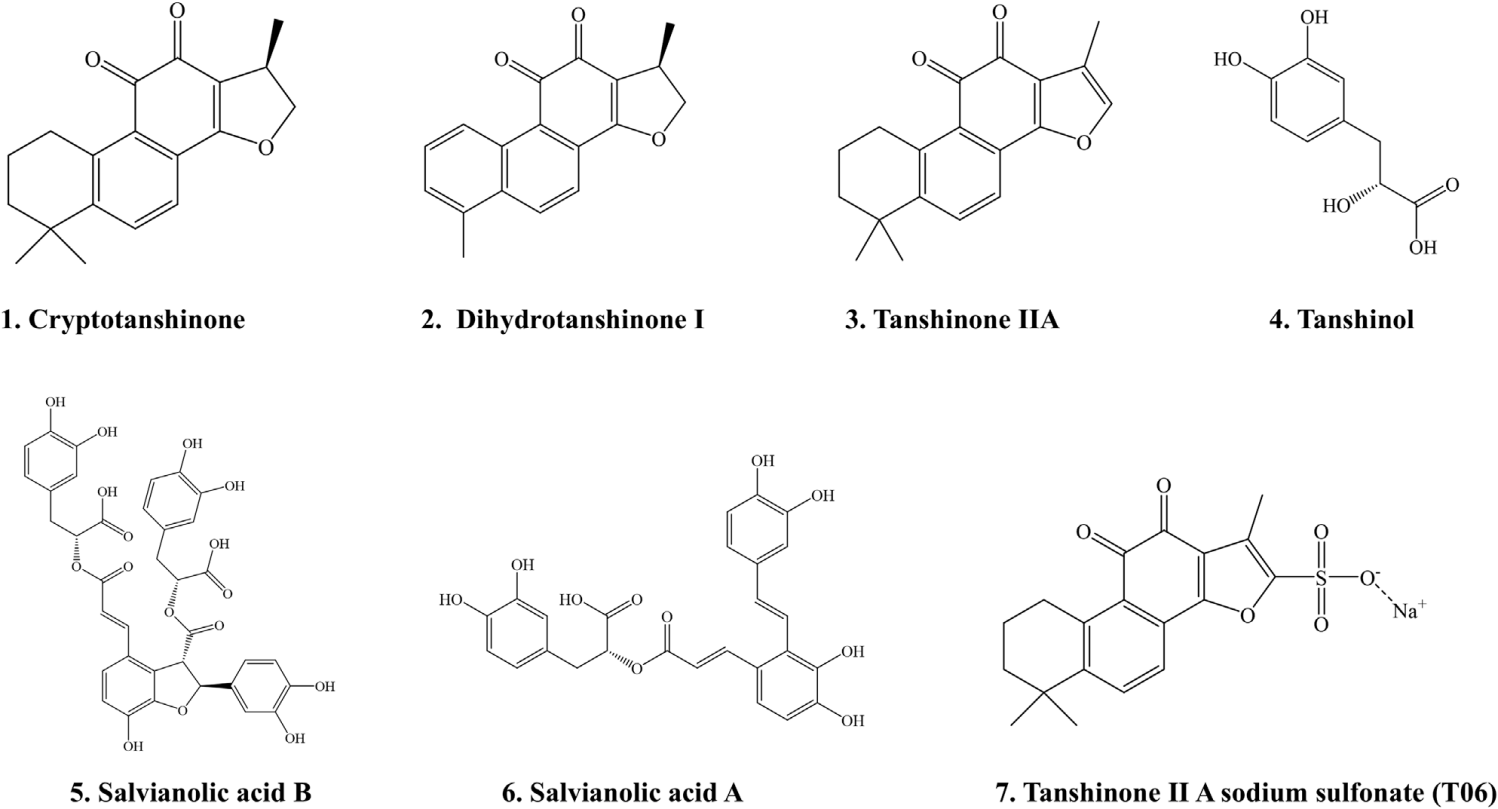
The chemical structures of *Salvia Miltiorrhiza* ingredients that are reported to mitigate osteoporosis in preclinical studies. 1. Cryptotanshinone; 2. Dihydrotanshinone Ⅰ; 3. Tanshinone IIA; 4. Tanshinol; 5. Salvianolic acid B; 6. Salvianolic acid A; 7. Tanshinone II A sodium sulfonate (T06).

The water-soluble phenolic acids were documented to exihibit anti-coagulant, anti-thrombotic (Tang et al., 2021), anti-inflammatory (Tang and Zhao, 2024) and anti-tumor (Yang et al., 2024) activities. The lipophilic diterpenoid tanshinones were reported to exhibit anti-oxidant (Jiang et al., 2023), anti-inflammatory (Tang and Zhao, 2024), anti-fibrotic (Zeng et al., 2024), anti-tumor (Yu et al., 2024), anti-osteoclastogenesis (Sudha et al., 2024) activities, and improve cognitive impairment (Liu et al., 2024b) and osteogenesis (Sudha et al., 2024). In the current review, we only listed the several chemical structures of SM ingredients that are reported to counteract osteoporosis in the preclinical studies (Figure 3).

## 6 Effects of *Salvia Miltiorrhiza* and its constituents on osteoporotic animal models

In order to study the effects of SM on osteoporosis, aqueous or ethanolic extracts of this herb as well as its hydrophilic and lipophilic constituents, such as salvianolic acid A, salvianolic acid B (Sal B), tanshinone IIA, cryptotanshinone, and tanshinones were used. Our group (Guo et al., 2014) had previously found that these countermeasures showed good anti-osteoporosis effects in different osteoporotic animal models based on the studies from 2003–2013. In the current review, we summarize the studies from 2014 to 2024 where SM itself and its active ingredients were used for osteoporosis management in different animals and cell models (Table 2).

**TABLE 2:** Effects of *Salvia Miltiorrhiza* and its constituents on bone remodeling in animal models.

Compound	Study design	Results	References
SM aqueous extracts (SMA)	Animals: SD rats aged 6 months Model: OVX-induced osteoporosis Treatment: 600 mg/kg/day of SMA for 12 weeks by gavage Negative control: 1% ethanol Positive control: 17 β-estradiol (1 mg/kg/day)	In LFS-fed OVX rats ↑Trabecular BMD ↓Weight gain, urinary Ca/Cr In HFS-fed OVX rats ↑Trabecular BMD, BV/TV ↓Tb.Sp ↑SOD, catalase (CAT) and GSH-Px; ↓MDA ↓Urinary Ca/Cr ↓Lipid deposition in livers	Dong et al. (2018)
SMA	Animals: Female SD rats (220 ± 10 g) Model: OVX-induced osteoporosis Treatment: 5 g/kg/day SMA for 14 weeks Negative control: Distilled water Positive control: Estradiol valerate (EV, 0.1 mg/kg/day	↑Femoral, lumbar, cancellous, plane and total BMD, trabecular area ratio; ultimate load, bending strength, and elastic modulus ↑OPG, β-catenin, p-GSK3β, IGF-1 and ALP in bone ↓DKK1, cathepsin K in bone ↓Body weight gain ↓Serum ALP and RANKL ↑Serum OPG	Liu et al. (2018)
SMA	Animals: Female ICR mice aged 7 weeks Model: OVX-induced osteoporosis Treatment: 50, 100, 200 mg/kg/day of SMA (by gavage) for 12 weeks Negative control: Phosphate-buffered-saline (PBS) Control: 50, 100, 200 mg/kg/day of SMA combined with liquified calcium (LCa) Positive control: 17 β-estradiol (0.1 mg/kg/day) or LCa (10 mL/kg/day)	All the dosages (50, 100, 200 mg/kg/day) of SMA were effective in attenuating bone loss ↑BMD, BV/TV, Tb.N, Tb.Pf, BS/BV ↑Serum OPG ↓Serum RANKL, OCN and BALP ↓mRNA expressions of cathepsin K, calcitonin receptor, TRAF6, NFTAc1 in bone	Park et al. (2017)
Tanshinol	Animals: Female SD rats aged 4 months Model: Glucocorticoid (GC) (prednisone, 5 mg/kg/d)-induced osteoporosis Treatment: Tanshinol (16 mg/kg/day) for 14 weeks Negative control: Distilled water Positive control: Resveratrol (5 mg/kg/day)	↑Body weights, Tb.N, Tb.Ar, Tb.Th, elastic load, bending energy ↓Tb.Sp, SMI ↑Bone formation ↓mRNA expressions of KLF15, PPARγ2, C/EBPα, aP2 and FoxO3a ↑mRNA expressions of Wnt, β-catenin, Axin2 and Gadd45a	Yang et al. (2018)
Tanshinol	Animals: Female SD rats aged 4–5 months Model: GC (prednisone, 5 mg/kg/day) induced osteoporosis Treatment: Tanshinol (25, 50 mg/kg/day) for 14 weeks Negative control: Distilled water Positive control: Calcitriol	↑BMD, Tb.Ar (25, 50 mg/kg/day) ↑BMC, Tb.N (25 mg/kg/day) ↓Tb.Sp (25 mg/kg/day) ↑Maximum load, breaking load, yield load, and bending energy (25, 50 mg/kg/day) ↑VEGF and TGF-β mRNA levels (25 mg/kg/day)	Chen et al. (2017a)
Tanshinol	Animals: Male SD rats aged 4 months Model: GC (prednisone, 5 mg/kg/day) induced osteoporosis Treatment: Tanshinol (25 mg/kg/day) for 14 weeks Negative control: Distilled water Positive control: No	↑BMD, BV/TV, Tb.Th, Tb.Ar, Ob.s/BS ↓Weight loss, Tb.Sp, Oc.s/BS and OC.N ↑Serum PINP ↓Serum β-CTX ↓Serum TXNIP, HIF-1α and VEGF ↓Bone TXNIP ↑Bone VEGF and β-Catenin ↑Maximum load, breaking load, stiffness	Lai et al. (2021)
Tanshinol	Animals: Female SD rats aged 4 months Model: GC (prednisone acetate, 5 mg/kg/day)-induced osteoporosis Treatment: 16 mg/kg/day tanshinol for 14 weeks by gavage Negative control: Distilled water Positive control: 5 mg/kg/day resveratrol	↑BV/TV, Tb.N, Tb.Th, fracture load, bending energy ↓Tb.Sp ↑Serum levels of OPG/RANKL, BALP and OCN ↑Bone mRNA levels of Col1α1, Runx2, Osx ↓Serum TRAP5b and bone OSCAR mRNA ↑Wnt pathway: Axin2, β-catenin ↓KLF15 in bone ↑Bone glutathione reductase activity (GSR) ↓Bone ROS, p-p66Shc/p66Shc	Yang et al. (2016)
Tanshinol	Animals: Female SD rats aged 4 months Experimental model: OVX-induced osteoporosis Treatment: 5 mg/kg/day tanshinol for 12 weeks by gavage Negative control: Corn oil Positive control: 25 μg/kg of β-estradiol	Corrected dyslipidemia (↑HDL-C; ↓TG, TC, LDL-C) ↓Bone turnover: serum levels of ALP, OCN, TRACP-5b ↓NF-κB pathway: p-IκBα/IκBα, p-p65/p65	Han and Wang (2017)
Tanshinol	Animals: Zebrafish Model: 10 µM Dex-induced osteoporosis zebrafish models Treatment: During 3- days’ postfertilization (dpf) and 9-dpf, tanshinol (0.5–50 µM) were added to egg water Negative control: 0.1% DMSO Positive control: 6 × 10^−6^ mg/mL rocalirol	↑Osteoblasts differentiation (1, 2.5, 5 µM tanshinol) ↑Osteoblasts differentiation (2.5, 5, 10, 50 µM tanshinol) ↑Bone mineralization (1, 2.5, 5 µM tanshinol) ↑Bone mineralization (1, 2.5, 5, 10, 50 µM tanshinol) ↑Osteoblast-specific mRNA expressions: Runx2a, OCN, ALP, and Osx (5 µM tanshinol) ↓ROS and MDA levels (5 µM tanshinol)	Luo et al. (2016a)
Sal B	Animals: Zebrafish Model: 10 µM Dex-induced osteoporosis zebrafish models Treatment: 0.2–10 µM Sal B for 6 days Negative control: 0.1% dimethylsulphoxide Positive control: Rocalirol 6 × 10^−6^ μg/mL	↑Osteoblasts differentiation (0.5, 1, 2 µM Sal B) ↑Osteoblasts differentiation (0.5, 1, 2, 4, 10 µM Sal B) ↑Bone mineralization (0.5, 1, 2 µM Sal B) ↑Bone mineralization (0.5, 1, 2, 4, 10 µM Sal B) ↑Osteoblast-specific mRNA expressions: Runx2a, OCN, ALP, and Osx (5 µM Sal B) ↓ROS and MDA levels (5 µM Sal B)	Luo et al. (2016b)
SM ethanolic extract (SME)	Animals: Female outbred ICR mice aged 6 weeks (OVX induced osteoporosis); female C57BL/6 mice aged 12 months (NM osteoporosis) Model: OVX-induced osteoporosis mice and NM mice Treatment: 50, 100, 200 mg/kg/day SME (6 times per week for 12 weeks by oral administration) Negative control: PBS Positive control: injections of 17 β-estradiol (0.1 mg/kg/day) 3 times per week	In OVX-induced osteoporosis mice ↑BMD (50, 200 mg/kg/day) ↑BV/TV, Tb.N (50, 100, 200 mg/kg/day) ↑Cs.Th (100, 200 mg/kg/day) ↓MMI (100 mg/kg/day) ↓Tb.Sp (50 mg/kg/day) ↓Serum levels of RANKL (100, 200 mg/kg/day), OCN (200 mg/kg/day), ALP (50, 100, 200 mg/kg/day) ↓mRNA expressions of cathepsin K (200 mg/kg/day), TRAF6 (50, 100, 200 mg/kg/day), NFATc1 (100, 200 mg/kg/day) In NM mice ↑BV/TV, Tb.Th (200 mg/kg/day) ↑Tb.pf (50, 100, 200 mg/kg/day) ↓Serum levels of RANKL, OCN (50, 100, 200 mg/kg/day), ALP (100, 200 mg/kg/day) ↑Serum OPG (50, 100, 200 mg/kg/day) ↓mRNA expressions of cathepsin K and TRAF6 (50, 100, 200 mg/kg/day)	Lee et al. (2020)
SME	Animals: Rats Model: LPS-induced dental alveolar bone resorption Treatment: 300 mg/kg/day of SME for 7 days by intraperitoneal injection Negative control: PBS Positive control: No	↓TRAP positive osteoclasts	Tsai et al. (2016)
Cryptotanshinone	Animals: Female SD rats aged 3 months Model: OVX-induced osteoporosis Treatment: 10 and 20 mg/kg/day of cryptotanshinone for 4 weeks Negative control: Not known Positive control: No	Both dosages (10 and 20 mg/kg/day) were effective in improvement of the following parameters ↑BMD, BMC, BV/TV, Tb.N, Tb.Th ↓Tb.Sp ↑Collagen formation (Masson staining) ↓Bone mass loss (HE stating)	Yang et al. (2022)
Tanshinone IIA	Animals: C57BL/6 mice Model: STZ-induced diabetic osteoporosis Treatment: Tanshinone IIA (10 and 30 mg/kg/day) for 8 weeks Negative control: Corn oil (used as drug vehicle) Positive control: Aliskiren (2 mg/kg)	↑BMD/TV, BV/TV (10 and 30 mg/kg/day) ↑Conn.D (30 mg/kg/day) ↓SMI (10 and 30 mg/kg/day) ↓serum and femur levels of angiotensin-II (10 and 30 mg/kg/day)	Zhang et al. (2020)
Tanshinone IIA	Animals: Female C57BL/6 mice aged 8 weeks Model: OVX-induced bone loss Treatment: Tanshinone IIA (10 mg/kg/day) for 6 weeks by injection Negative control: Not known Positive control: No	↑BMD, BS/TV, BV/TV, Tb.N ↓Tb.pf	Cheng et al. (2018)
Tanshinone IIA	Animals: Female SD rats aged 3 months Model: OVX-induced alveolar bone loss Treatment: Tanshinone IIA (10 mg/kg/day) for 2 weeks by tail vein injection Negative control: Not known Positive control: No	↑BV/TV, Tb.N, Tb.Th ↓Tb.Sp	Wang et al. (2019a)
Dihydrotanshinone I (DTI)	Animals: 12-weeks-old mice Model: OVX mice Treatment: DTI (10 mg/kg/2 days) for 6 weeks by intraperitoneal injection Negative control: No treatment Positive control: No	↑BV/TV, Tb.Th, BMD, Tb.N ↓Tb.Sp ↓Osteoclast numbers and surface area in bone slices (TRAcP staining)	Ma et al. (2023)
Tanshinone IIA Sulfonic Sodium (T06)	Animals: 12-weeks-old mice Model: OVX mice Treatment: 40 mg/kg/day of T06 for 3 months Negative control: No treatment Positive control: No	↑Tb.N, BMD by 35%, osteoblast numbers and plasma PINP ↓Tb.Sp, plasma CTX-1	Panwar et al. (2017)

For the animal studies, ovariectomized (OVX) and naturally menopaused (NM) mice or rats were used to mimic estrogen deficiency-induced osteoporosis. Prednisone, dexamethasone (Dex)-treated mice, rats or zebrafish were used to establish glucocorticoid (GC)-induced osteoporotic animal models. Streptozotocin (STZ) and high-fat diet-treated mice or rats were used to make diabetic osteoporosis rodent models. To determine the effects of SM on bone microstructure, dual-X-ray absorptiometry (DXA) was used in the quantification of BMD, and bone mineral content (BMC). Micro-computed tomography (μ-CT) was used to analyze 3D and 2D bone microstructures, including BMD, BMC, bone volume over total volume (BV/TV), trabecular thickness (Tb.Th), trabecular separation (Tb.Sp), trabecular number (Tb.N), and structural model index (SMI). Three-point bending assay was used to analyze bone biomechanical strength. Besides, bone histomorphometry and bone remodeling markers were employed as tools for evaluating both bone quality and turnover. Bone remodeling markers were primarily divided into bone resorption parameters (RANKL, OPG, RANKL/OPG, CTX, TRAP) and bone formation parameters (PINP, OCN, BALP, E_2_) (Table 2).

In the present study, we firstly review experiments using SM aqueous extracts (SMA) and their constituents. The doses of SMA used ranged from 50 mg/kg/day to 5 g/kg/day, and the treatment durations were 12 ∼14 weeks for the OVX-induced osteoporotic rats. Together, these studies indicated that SMA reversed estrogen deficiency-induced osteoporosis by OVX (Dong et al., 2018; Liu et al., 2018; Park et al., 2017). The underlying mechanisms were related to attenuate lipid accumulation and oxidative stress levels (Dong et al., 2018), favor OPG/wingless-related integration site (Wnt)/β-catenin-induced osteoblast differentiation, block RANKL-induced osteoclast differentiation via down-regulating the expressions of tumor necrosis factor receptor-associated factor 6 (TRAF6), nuclear factor of activated T cells 1 (NFTAc1), cathepsin K and calcitonin (Dong et al., 2018; Liu et al., 2018).

Tanshinol, also recognized as Danshensu, a water-soluble component in *S. Miltiorrhiza*. Studies have documented that tanshinol at concentration of 16, 25 and 50 mg/kg/day and therapeutic duration for 14 weeks attenuated GC-induced bone loss via promoting osteogenesis through the following pathways (Yang et al., 2018; Yang et al., 2016; Chen et al., 2017a; Lai et al., 2021): 1) Generally, osteogenesis is mainly mediated by the Wnt/β-catenin signaling pathway, while vascular endothelial growth factors (VEGFs) are key regulators in the cascade of angiogenesis and osteogenesis. The administration of GC to rat upregulated thioredoxin interacting protein (TXNIP) and kruppel-like factor (KLF15), which leads to an inhibition of the Wnt and VEGF pathways (Mo et al., 2021). Tanshinol was reported to promote angiogenesis and osteogenesis via downregulation of the TXNIP/KLF15 signaling and upregulation of the Wnt and VEGF pathways to attenuate bone loss in mice exposed to GC (Yang et al., 2016; Chen et al., 2017a; Lai et al., 2021). 2) It is well-known that both osteogenesis and bone marrow adipogenesis are regulated by the Wnt pathway in bone marrow mesenchymal stromal cells (BMSCs) (Karthik and Guntur, 2021). And KLF15 stimulated by GC can trigger the upregulation of peroxisome proliferator-activated receptor-γ (PPARγ), enhancer-binding protein α (C/EBPα) and adipocyte fatty acid binding protein 2 (aP2), an adipocyte-specific gene), thusly contributing to adipogenesis. Moreover, KLF15 triggers expression of forkhead box O3 (FoxO3a), leading to an inhibition of osteoblast differentiation via downregulation of the Wnt pathway (Yang et al., 2018). Interestingly, tanshinol was shown to counteract GC-induced bone loss by inhibiting marrow adiposity through attenuation of the KLF15/PPARγ2/C/EBP/FoxO3a pathway and upregulation of the Wnt/β-catenin signaling in rats (Yang et al., 2018).

Recent interest has paid attentions to the contribution of free radicals in bone remodeling (Leon-Reyes et al., 2023). During the development of osteoporosis, over-production of reactive oxygen species (ROS) causes bone loss and microstructure disruption (Fan et al., 2023). Several lines of evidence revealed that GC exposure leads to the over-accumulation of intracellular ROS. Interestingly, both tanshinol (Yang et al., 2016; Luo et al., 2016a) and Sal B (Luo et al., 2016b) could reverse GC-induced inhibition of bone formation via inhibiting ROS over-production. Using Dex-treated larval zebrafish, both Sal B (2 µM for 6 days) and tanshinol (1–50 µM) were reported to promote osteogenesis by stimulating the expressions of osteoblast-specific genes (runt-related transcription factor 2a [Runx2a], OCN, ALP, and osterix [Osx]) via inhibiting ROS over-production (Luo et al., 2016b; Luo et al., 2016a). In addition, phosphorylation of p66^Shc^ is an adapter protein that is recognized for its role in enhancing mitochondrial ROS production and promoting apoptosis (Haslem et al., 2022). Yang and colleagues revealed that tanshinol (16 mg/kg/day for 6 weeks) inhibited bone loss by attenuating oxidative stress via regulation of the ROS/p66Shc pathway in GC-induced osteoporotic rats (Yang et al., 2016).

Using OVX-induced osteoporosis mice, tanshinol (5 mg/kg/day treated for 12 weeks) was reported to attenuate bone loss by blocking nuclear factor-kappa B (NF-κB) pathway (Han and Wang, 2017). In addition, Sal B (40 mg/kg/day for 3 weeks) was reported to promote bone formation in rats with tibia fracture (He and Shen, 2014)

The following studies refer to SM ethanolic extracts (SME) and its lipophilic constituents. Lee et al. showed that SME (50–200 mg/kg) ameliorated trabecular bone loss both in OVX mice and naturally menopaused (NM) mice. Further investigation showed that SME was effective in correcting aberrant levels of RANKL, OCN, and BALP, and suppressing the gene expressions of TRAF6, NFATc1 and cathepsin K to inhibit osteoclast differentiation and bone resorption (Lee et al., 2020). Tsai et al. found that 95% SME (the major components were 30% tanshinone IIA, 5.8% cryptotanshinone, and 2.8% tanshinone I) attenuated dental alveolar bone resorption caused by lipopolysaccharide in rats, as evidenced by decreased numbers and percentage of osteoclasts (Tsai et al., 2016).

Lipophilic compounds, such as cryptotanshinone (Yang et al., 2022), tanshinone IIA (Wang et al., 2019a; Cheng et al., 2018) and dihydrotanshinone I (Ma et al., 2023), were shown to prevent trabecular bone loss in OVX rats. Oral administration of cryptotanshinone (10 and 20 mg/kg/day for 4 weeks) to OVX rats attenuated bone microarchitectural deterioration and prevented bone loss (Yang et al., 2022). Dihydrotanshinone I at a dose of 10 mg/kg for a duration of 6 weeks reversed bone loss in OVX mice via inhibiting the NF-κB/extracellular signal regulated kinase (ERK)/NFATc1 signaling pathways (Ma et al., 2023). Tanshinone IIA (Wang et al., 2019a; Cheng et al., 2018) at concentration of 10–40 mg/kg/day for 2–12 weeks alleviated OVX- or STZ-induced bone loss. The underlying mechanism was related to inhibiting angiotensin-II expression (Zhang et al., 2020), attenuating osteoclastogenesis by inactivating the NF-κB/protein kinase B (Akt) signaling pathways (Cheng et al., 2018), and up-regulating phosphoglycerate dehydrogenase (PHGDH) (Wang et al., 2019a). Tanshinone IIA Sulfonic Sodium (T06) at 40 mg/kg/day for 3 months reduced plasma CTX-1 by 20%, increased osteoblast numbers and plasma PINP by ∼28%, and increased BMD by 35% in OVX-induced osteoporotic animals. And further study showed that T06 is a potent cathepsin K inhibitor, as it selectively inhibited collagen degradation induced by cathepsin K (Panwar et al., 2017).

In summary, *in vivo* studies clearly reveal that SM and its active components preserve bone quality in various osteoporotic rodent models. The aqueous extracts of SM and their constituents (tanshinol, Sal B) attenuate bone loss via promoting angiogenesis and inhibiting ROS over-generation. While ethanolic components (tanshinones) exhibit inhibitory effects on osteoclastogenesis and bone resorption.

## 7 *Salvia Miltiorrhiza* and its constituents in osteoporotic cell models

### 7.1 Actions and mechanisms of *Salvia Miltiorrhiza* and its constituents on osteoclastogenesis and bone resorption

Osteoclasts are multinucleated cells that derived from monocyte/macrophage-lineage cells, attaching tightly to bone surfaces to secrete acids and proteases such as cathepsin K, finally leading to dissolution of bone mineral and degradation of bone organic matrix. Macrophage colony-stimulating factor (M-CSF) and RANKL secreted by osteoblasts are pivotal cytokines critically involved in orchestrating osteoclastogenesis, including the intricate processes of osteoclast formation, differentiation, and resorption. Osteoclast precursors express RANK, and differentiate into osteoclasts to perform bone resorption in the presence of M-CSF and RANKL secreted by osteoblasts (Foger-Samwald et al., 2022; Udagawa et al., 2021). Thus, inhibition of excessive osteoclast differentiation stands as a promising therapeutic strategy aimed at mitigating the onset and progression of osteoporosis. Several *in vitro* studies investigated the effects of SM and its ingredients on osteoclasts differentiation using M-CSF and/or RANKL stimulated osteoclast precursors, including bone marrow derived macrophages (BMMS), and RAW 264.7 cells (Table 3).

**TABLE 3:** Effects and mechanisms of SM and its constituents on osteoclasts and osteoblasts.

Compounds	Osteoclast/Osteoblast and intervention	Results	References
Osteoclasts differentiation and osteoclastic bone resorption			
Cryptotanshinone (CPT)	Cell: RAW 264.7 Model: RANKL-induced osteoclast differentiation Treatment: 1 and 10 µM of CPT for 1 h	↓Osteoclast differentiation, TRAP activity ↓NFATc1, c-fos, cathepsin K	Yang et al. (2022)
Tanshinone IIA	Cell: RAW264.7 Model: RANKL and M-CSF-induced osteoclast differentiation Treatment: Tanshinone IIA (1, 2, 5 μg/mL) for 7 days	↓TRAP^+^ cells, resorption pits, NFATc1, TRAP, MMP-9, cathepsin K, CTR, TRAF6, p-p65/p65, p-p50/p50, p-IκBα/IκBα, p-ERK/ERK, p-JNK/JNK, and p-P38/P38, p-Akt/Akt, p-c-fos/c-fos	Cheng et al. (2018)
CPT	Cell: BMMS Model: RANKL-induced osteoclast differentiation Treatment: 0.1–40 µM of CPT	↓Osteoclast differentiation, TRAP activity, TRAP^+^ osteoclasts, resorption area ↓TRAF6, NFATc1, c-fos, p-ERK/ERK, p-P65/P65	Wang et al. (2019c)
Tanshinones	Model: Cathepsin K mediated degradation of soluble collagen Treatment: Tanshinones (10 µM)	↓Degradation of soluble collagen	Panwar et al. (2018)
Dihydrotanshinone I (DTI)	Model: Cathepsin K mediated degradation of soluble and insoluble collagen Treatment: DTI (0.5–50 µM)	↓Degradation soluble collagen and gelatin ↓Degradation of insoluble collagen fiber ↓TRAP^+^ osteoclasts and resorption pits on bone surfaces	Panwar et al. (2016)
Dihydrotanshinone I (DTI)	Cell: RAW264.7 cells Model: RANKL-induced osteoclastogenesis Treatment: 1, 2 µM of DTI	↓Osteoclasts area, osteoclasts numbers ↓2 μM of DTI significantly inhibited the expressions of osteoclast-specific markers (Acp5, c-fos, NFATc1, CTSK, Atp6vod2), and phosphorylation of ERK and JNK, nuclear heterotopia of p65, degradation of IκBα protein	Ma et al. (2023)
Tanshinone IIA Sulfonic Sodium (T06)	Model: Cathepsin K-mediated degradation of soluble and insoluble collagen Treatment: T06 (0.5–50 µM)	↓Collagen degradation	Panwar et al. (2017)
Osteoblasts differentiation and osteoblast-mediated osteogenesis			
SM aqueous extract (SMA)	Cell: Pre-osteoblast MG63 cells Model: H_2_O_2_ -treated cells Treatment: SMA (5, 10, 15 μg/mL) for 24 h	↑Cell viability ↑ALP activity ↓Intracellular ROS levels	Dong et al. (2018)
Tanshinol	Cell: MG cells and EA cells Model: Dex-treated cells Treatment: Tanshinol (dose was not mentioned) for 24 h	↑Migration and tube formation of EA cells ↑VEGFR2 and β-catenin proteins in MG cells and EA cells Supernatants from EA cells promoted the expressions of VEGFR2 and β-catenin in MG cells	Lai et al. (2021)
Tanshinol	Cell: Pluripotent mesenchymal precursor C2C12 cells and pre-osteoblastic MC3T3-E1 cells Model: Dex (1 µM) and/or RU486 (GC receptor antagonist) and/or siRNA-KLF15 -treated cells Treatment: Tan (1 µM)	↑Osteoblastic differentiation (ALP staining) ↑Bone formation (Alizarin Red S) ↓KLF15 mRNA	Yang et al. (2016)
Tanshinol	Cell: MC3T3-E1 Model: Normal cultured MC3T3-E1 Treatment: 0–400 μg/mL of tanshinol for 4 days or 48 h	↑Osteoblast viability and ALP activity ↓Apoptosis (↑Bcl-2/Bax; ↓apoptotic area) ↑mRNA and protein expressions of Col1A1, Runx2 and OCN	Han and Wang (2017)
Sal B and Tanshinol	Cell: BMSCs Model: Osteogenic induction of MSCs (OB-IN) Treatment: 0.05, 0.5, 2.5 µM of Sal B and 2 µM of Danshensu	↑ALP activity and OCN ↑OPG, OPG/RANKL ↑NO secretion ↓RANKL	Zhang et al. (2017)
Sal B	Cell: Primary osteoblasts were isolated from the skull of 3-day-old SD rat Model: SMG-induced osteoblasts	↑Osteoblast proliferation, ALP activity ↓Apoptosis ↓Oxidative stress (↑SOD, CAT, LDH, GSH-Px; ↓MDA) ↑Osteogenesis: Runx2, Osx, OPN, ALP, Col-I ↑Antioxidant: Nrf2, HO-1	Wang et al. (2018)
Sal B	Cell: Primary osteoblasts were isolated from the skull of SD rats Model: 10^–6^ μg/mL prednisolone acetate (PA) stimulated osteoblast Treatment: Sal B (dose was not provided)	↑ALP activity ↑Antioxidant: Nrf2 ↑Osteoblasts differentiation: Runx2 and Osx ↑Osteogenesis: Col-I, IGF-I and OCN	Qiao et al. (2019)
Sal B	Cell: Human mesenchymal stem cells (hMSCs) Model: Osteogenic induced medium (OIM) induced hMSCs Treatment: Sal B (0.1, 0.5, 1 and 5 µM)	↑ALP activity, mineralization of hMSCs ↑Runx2, Osx, OPN, OCN, p-ERK/ERK	Xu et al. (2014)
Sal B	Cell: MC3T3-E1 and 3T3-L1 Model: TAZ knock-down cells Treatment: Sal B (0.1, 1 and 10 μmol/L) for 3, 7 or 14 days	↑Osteogenesis (TAZ, Runx2 and OCN) in MC3T3-E1 cells ↓Adipogenesis (↓C/EBPβ and PPARγ; ↑TAZ) in 3T3-L1 cells ↑p-ERK/ERK in both cell lines TAZ knock-down counteracts upregulation of Runx2 and OCN expressions and downregulation of C/EBPβ and PPARγ expressions in 3T3-L1 cells in response to Sal B treatment	Wang et al. (2019b)
Tanshinone IIA (Tan IIA)	Cell: Human periodontal ligament stem cells (hPDLSCs) Model: Normal cultured hPDLSCs Treatment: Tan IIA (2.5 and 5 µM)	↑Proliferation of hPDLSCs ↑Osteoblasts differentiation of hPDLSCs: Alizarin red-positive calcium deposition; mRNA and protein expressions of ALP, OPN, OCN, Runx2 ↓Adipogenic differentiation of hPDLSCs: mRNA expressions of LPL and PPARγ; Oil red O-positive cytoplasmic lipid accumulation ↓Apoptosis of hPDLSCs: C-PARP, Ccaspase-3	Liu et al. (2019)
Tan IIA	Cell: Osteoblastic MC3T3-E1 cells Model: Dex (1 µM)-induced cell apoptosis Treatment: Tan IIA (1 µM)	↑Cell viability Attenuated Dex-induced apoptosis ↓Cytosol cytochrome C, Bax, activity of caspase-9/-3 ↑Bcl-2 ↓ROS, Nox4	Li et al. (2015)
Tan IIA	Cell: BMSCs isolated from the mandible of OVX rats Model: OVX-induced BMSC senescence Treatment: Tan IIA (20 µM)	↑Proliferation of BMSCs ↑Protein levels of BMSCs: Nanog, SOX2 and octamer-binding transcription factor 4 (OCT4) ↓mRNA expression of PHGDH ↓BMSCs senescence: SA-β-gal positive cells	Wang et al. (2019a)
Tan IIA	Cell: Mouse BM-MSCs Model: Osteogenic differentiation of mouse BM-MSCs Treatment: Tan IIA (1, 5, 10, and 20 µM)	1, 5 µM of Tan IIA ↑ALP activity, calcium concentration ↑Gene expressions of OPN, Col-I, OPG, Runx2, BMP-4, β-catenin, and Cyclin D1 ↓Gene expressions of RANKL 10 µM of Tan IIA: No changes 20 µM of Tan IIA ↓ALP activity, calcium concentration ↓Gene expressions of OPN, Col-I, OPG, Runx2, BMP-4, β-catenin, and Cyclin D1 ↑Gene expression of RANKL	Qian et al. (2015)

The lipophilic tanshinones, including cryptotanshinone (CPT) (Wang et al., 2019c; Yang et al., 2022), tanshinone IIA (Cheng et al., 2018), dihydrotanshinone I (Ma et al., 2023; Panwar et al., 2016), at concentrations of 0.1–40 µM (as shown in Table 3), were reported to inhibit osteoclast differentiation and bone resorption. It is well established that RANKL binds to its receptor RANK, then recruits TRAF6 and activates the downstream signaling of AKT, ERK, NF-κB, c-fos and NFATc1 to regulate osteoclast-specific genes, including TRAP, H^+^-ATPase, Atp6v0d2, Oscar, and cathepsin K, finally leading to osteoclastic bone resorption (Hodge et al., 2011). Cryptotanshinone at concentrations from 0.1 to 40 µM was reported to dose-dependently decrease the TRAP-positive osteoclasts and reduce resorption pits in BMMs (Wang et al., 2019c). In another study, cryptotanshinone (0.1 and 10 µM) was demonstrated to decrease osteoclasts formation (Yang et al., 2022) through downregulating the expressions of osteoclastogenesis-related genes such as TRAF6, c-Fos, NFATc1, cathepsin K via blocking the ERK/NF-κB pathway (Wang et al., 2019c; Yang et al., 2022). Tanshinone IIA (at 1, 2, 5 μg/mL) was reported to dose-dependently attenuate RANKL-induced osteoclastogenesis and osteoclastic bone resorption through inhibiting the expressions of TRAP, cathepsin K, TRAF6, matrix metalloproteinases (MMP)-9, CTR, and NFATc1 via inhibiting the NF-κB/Akt/MAPK/c-fos signaling pathway (Cheng et al., 2018). Dihydrotanshinone I (2 µM) was shown to attenuate RANKL-induced osteoclastogenesis targeting the ERK, NF-κB and NFATc1 signaling pathways (Ma et al., 2023). These findings put tanshinones into anti-RANKL agents with the functions of attenuating bone resorption through the inhibition of osteoclastogenesis.

RANK-RANKL signaling also regulates cathepsin K expression. It is well known that Cathepsin K, the primary protease within osteoclasts, plays an exclusive role in the degradation of type I collagen (Col-I), which constitutes approximately 90% of the organic bone matrix (Costa et al., 2011). Therefore, cathepsin K emerges as a promising candidate for therapeutic intervention in combating osteoporosis. Dieter Brömme and his team successively proved the potential of lipophilic tanshinones as a potent cathepsin K inhibitor (Panwar et al., 2017; Panwar et al., 2018). They found that dihydrotanshinone I, a lipophilic abietane diterpenoids from *S. Miltiorrhiza*, inhibited cathepsin K induced insoluble collagen fiber degradation (Panwar et al., 2016). They found that tanshinone IIA Sulfonic Sodium (T06) with an IC50 value of 2.7 ± 0.2 µM exhibited an inhibition of cathepsin K activity by inhibiting cathepsin K induced insoluble collagen fiber degradation (Panwar et al., 2017). They further reported that SM herbal extracts and its 12 kinds of tanshinones (T02, T05, T06, T07, T08, T09, T11, T12, T17, T20, T23 and T27 at 10 µM) exhibited inhibition of cathepsin K mediated degradation of soluble collagen (Panwar et al., 2018). These findings put these tanshinones into anti-cathepsin K agents.

In summary, *in vitro* evidence supports the notion that lipophilic tanshinones extracts from SM, including cryptotanshinone, tanshinone IIA, dihydrotanshinone I, inhibits RANKL-induced osteoclast proliferation and differentiation targeting the MAPK/NF-κB/NFATc1 signaling pathway, thusly reducing osteoclastic bone resorption. And some of tanshinones are documented to be effective inhibitors of cathepsin K, as they inhibited cathepsin K-induced degradation of collagen in bone matrix. These findings help to understand the molecular mechanisms of anti-antiresorptive and anti-osteoporotic actions of SM, and put tanshinones into the same mechanistic class as potential anti-RANKL and anti-cathepsin K agents.

### 7.2 Actions and mechanisms of *Salvia Miltiorrhiza* and its constituents on osteogenesis/bone formation

Osteoblasts, constituting 4%–6% of the total resident bone cells, derive from pluripotent mesenchymal stem cells (MSCs). Mature osteoblasts reside in close proximity to the bone surface, where they orchestrate bone formation via synthesis and secretion of bone matrix proteins (Col-I) to guide mineralization of bone meshwork. And upon completion of bone formation, most of mature osteoblasts undergoes differentiation into osteocytes, which subsequently become encapsulated within the newly formed bone matrix (Veis and O'Brien, 2023; Foger-Samwald et al., 2022).

To investigate the actions of SM and its ingredients on osteogenesis, various osteoblast precursors were used, including MC3T3-E1 cells, MG cells, MG63 cells, pluripotent mesenchymal precursor C2C12 cells, human periodontal ligament stem cells (hPDLSCs), BMSCs and human mesenchymal stem cells (hMSCs). The modeling approaches to osteoblasts include GC (Dex, PA), H_2_O_2_, simulated microgravity condition (SMG) and OVX-induced inhibition of osteogenic differentiation and osteogenesis (Table 3). It should be noted that both water-soluble substance [mainly Sal B, tanshinol (Danshensu)] and ethanol-soluble ingredients (mainly tanshinone IIA) exerting positive effects on osteoblast differentiation and osteogenesis (Table 3).

To evaluate the actions of SM on osteoblastic bone formation, the following makers were evaluated, including Osx, Runx2, Col-I, insulin-like growth factor 1 (IGF-I), OCN, osteopontin (OPN), and osteonectin. Of these, Osx and Runx2 are related to the development and differentiation of osteoblasts (Nakashima et al., 2002; Vimalraj et al., 2015). Col-I, OPN, OCN, and IGF-I are involved in the production of bone extracellular matrix. Col-I is the most important fibrous collagen component, while OCN and OPN are major non-collagenous proteins in the bone matrix. OCN binds strongly to Col-I and hydroxyapatite, playing a crucial role in the mineralization of extracellular matrix (Zhu et al., 2020), while IGF-1 released from bone matrix stimulates MSCs differentiation to osteoblasts (Xian et al., 2012), thereby promoting bone matrix synthesis (Crane and Cao, 2014). It has been demonstrated that tanshinone IIA at concentration from 1 to 5 µM (Liu et al., 2019), Sal B from 0.1 to 20 µM (Xu et al., 2014; Wang et al., 2018; Zhang et al., 2017), and tanshinol at concentrations from 1 to 5 µM (Yang et al., 2016; Zhang et al., 2017) increased genes expressions of Runx2, Osx, OCN, and OPN under different conditions.

The bone morphogenic proteins (BMPs) and Wnt/β-catenin are two major signaling pathways responsible for osteoblast differentiation and osteogenesis by directly triggering Runx2 expression (Gaur et al., 2005; Teufel and Hartmann, 2019). One study demonstrated that pre-treatment with tanshinone IIA (1, 5 µM for 7 or 24 days) upregulated ALP expression and calcium content in bone marrow-derived MSCs (BM-MSCs) by upregulating osteogenesis-related gene expressions (Runx2, Col-I, OPN, OPG) targeting the BMP and Wnt/β-catenin signaling pathways (Qian et al., 2015). Recently, growing attentions have been paid to an endosteal type H capillary, which couples angiogenesis with osteogenesis (Kusumbe et al., 2014). A crosstalk between the VEGF signaling and Wnt pathway was proposed to investigate the links between vascular cells and osteoblasts. Tanshinol was reported to attenuate the inhibition of migration ability and tube formation in Dex-stimulated EA cells (human umbilical vein endothelial cells), thereby contributing to bone formation in MG cells by upregulating the expressions of VEGFR2 and β-catenin (Lai et al., 2021). Further study found that tanshinol (1 µM) rescued Dex-induced inhibition of osteoblastic differentiation and bone formation in C2C12 cells and MC3T3-E1 cells via regulating KLF15 expression (Yang et al., 2016). It is well known that administration of Dex upregulates KLF15 expression, thusly leading to an inhibition of the Wnt and VEGF pathways [64].

It is known that ERKs activation stimulates the phosphorylation and transcription of Runx2 (Ge et al., 2009). Sal B at 0.1–5 µM increased ALP activity and upregulated the expressions of osteogenesis-related genes (Runx2, Osx, OCN, OPN, BSP) through activating the ERK signaling pathway to promote mineralization of hMSCs (Xu et al., 2014). Furthermore, it is well accepted that ERK triggers the upregulation of TAZ (transcriptional co-activator with PDZ-binding motif), and further stimulates Runx2-mediated osteogenic differentiation (Byun et al., 2014), and inhibits PPARγ-induced adipogenesis (El Ouarrat et al., 2020). Sal B (0.1 or 1 µM) was found to reduce adipogenesis of 3T3-L1 cells through downregulation of C/EBPβ and PPARγ expressions, and facilitate osteogenesis of MC3T3-E1 cells through upregulation of Runx2 and OCN targeting TAZ via activating the MEK-ERK pathway (Wang et al., 2019b). hPDLSCs are postnatal stem cells that can be differentiated into adipocytes, osteoblasts and odontoblasts. It is reported that tanshinone IIA (2.5 and 5 µM for 48 h) facilitated osteogenic differentiation of hPDLSC through upregulating the expressions of Runx2, OCN, OPN, and ALP via activating the ERK1/2 signaling, while inhibited adipose differentiation of hPDLSC via down-regulating the PPARγ pathway (Liu et al., 2019).

Excessive ROS production disturbs redox homeostasis, leading to an inhibition of osteoblast proliferation and differentiation, and an acceleration of osteoblast apoptosis, thereby jeopardizing bone formation (Leon-Reyes et al., 2023). SMA, Sal B and tanshinone IIA were reported to exhibit an anti-oxidative effect to promote osteogenesis (Dong et al., 2018; Li et al., 2015; Qiao et al., 2019; Wang et al., 2018). SMA at concentrations of 10 and 15 μg/mL was reported to promote proliferation (cell viability) and differentiation (ALP activity) of pre-osteoblasts through reducing cellular ROS levels in H_2_O_2_-treated MG63 cells (Dong et al., 2018). It is known that NADPH oxidase 4 (Nox4) is one of major sources of ROS production (Kuroda et al., 2014). Tanshinone IIA at 1 µM was reported to block Dex-induced osteoblasts apoptosis through inhibiting Nox4-derived ROS production (Li et al., 2015). In addition, p66Shc is an adapter protein that is recognized for its role in enhancing mitochondrial ROS production and promoting apoptosis [68]. Tanshinol (1 µM) was reported to attenuate Dex-induced ROS over-production and osteoblasts apoptosis via regulation of phosphorylation of p66Shc (Yang et al., 2016).

Moreover, nuclear factor erythroid 2-related factor 2 (Nrf2) is one of the best characterized anti-oxidant cascades, functioning to regulate antioxidant response and protect osteoblasts from excessive oxidative stress (Wang et al., 2023). After activation, Nrf2 is translocated to the nucleus and binds to anti-oxidant response element (ARE), leading to activation of the antioxidant enzyme heme oxygenase 1 (HO-1) and consequent upregulation of Runx2 expression (Fan et al., 2023). Sal B at concentrations of 25–55 μg/mL was reported to increase osteoblasts activity and upregulate expressions of Runx2, Osx, OPN, ALP, and Col-I under simulated microgravity condition (SMG) through inhibiting oxidative stress via regulating the Nrf2/ARE/HO-1 signaling pathway (Wang et al., 2018). In another study, Sal B was reported to ameliorate Dex-induced osteoblasts activity through upregulating bone formation related gene expressions (Col-I, IGF-I, OCN, Runx2 and Osx) via regulating HO-1 expression (Qiao et al., 2019).

Recently, the discovery of senescent cells’ causative role in age-related bone loss has garnered significant attention among scientists and clinicians (Foger-Samwald et al., 2022). Indeed, BMSCs isolated from OVX rat exhibited early signs of senescence (Wang et al., 2019a). Interestingly, tanshinone IIA at concentration of 20 µM could suppress OVX-induced BMSCs senescence through up-regulating PHGDH (Wang et al., 2019a). It is known that PHGDH is negatively associated cell senescence (Wu et al., 2023). One study reported that tanshinol (0–400 μg/mL) dose-dependently increased ALP activity, upregulated osteogenesis-related genes expressions of CollA1, Runx2, and OCN, while inhibited apoptosis (increase of Bcl-2 and Bcl-2/Bax; decrease of Bax) in MC3T3-E1 cells (Han and Wang, 2017).

Nitric oxide (NO) is produced in both osteoblasts and osteoclasts. And NO donors are proposed to promote bone formation and accelerate bone healing process, as well as decrease osteoporotic fractures (Yousefzadeh et al., 2021). Indeed, NO donor sodium nitroprusside was able to increase the OPG/RANKL ratio in MSCs (Fan et al., 2004), thereby preventing osteoclastic bone resorption and promoting osteogenesis (Udagawa et al., 2021). In addition, NO donor nitroglycerin mitigated GC-induced bone loss in rats (Li et al., 2007). It is documented that NO bioavailability was decreased in the bones of both OVX rats and postmenopausal women, which may be one of the primary cause underlying osteoporosis (Yousefzadeh et al., 2022). Intriguingly, Sal B (0.05, 2, 5 µM) and tanshinol (2 µM) were reported to promote NO secretion, thusly increase the OPG/RANKL ratio, leading to the osteogenic differentiation of rat BMSCs (OCN and ALP activity) (Zhang et al., 2017).

Overall, both water-soluble substance (mainly Sal B and tanshinol) and ethanol-soluble tanshinone IIA were shown to promote osteoblastogenesis and osteogenesis. The underlying mechanisms were related to inhibiting ROS over-production via regulation of the Nrf2/ARE/HO-1 pathway, boosting the coupling between angiogenesis and osteogenesis via regulation of the Wnt/β-catenin/VEGF pathways, promoting osteogenesis via activation of the ERK/TAZ pathway, and inhibiting adipose differentiation of osteoblast precursors via downregulation of the PPARγ pathway.

## 8 Conclusion

**FIGURE 4:**
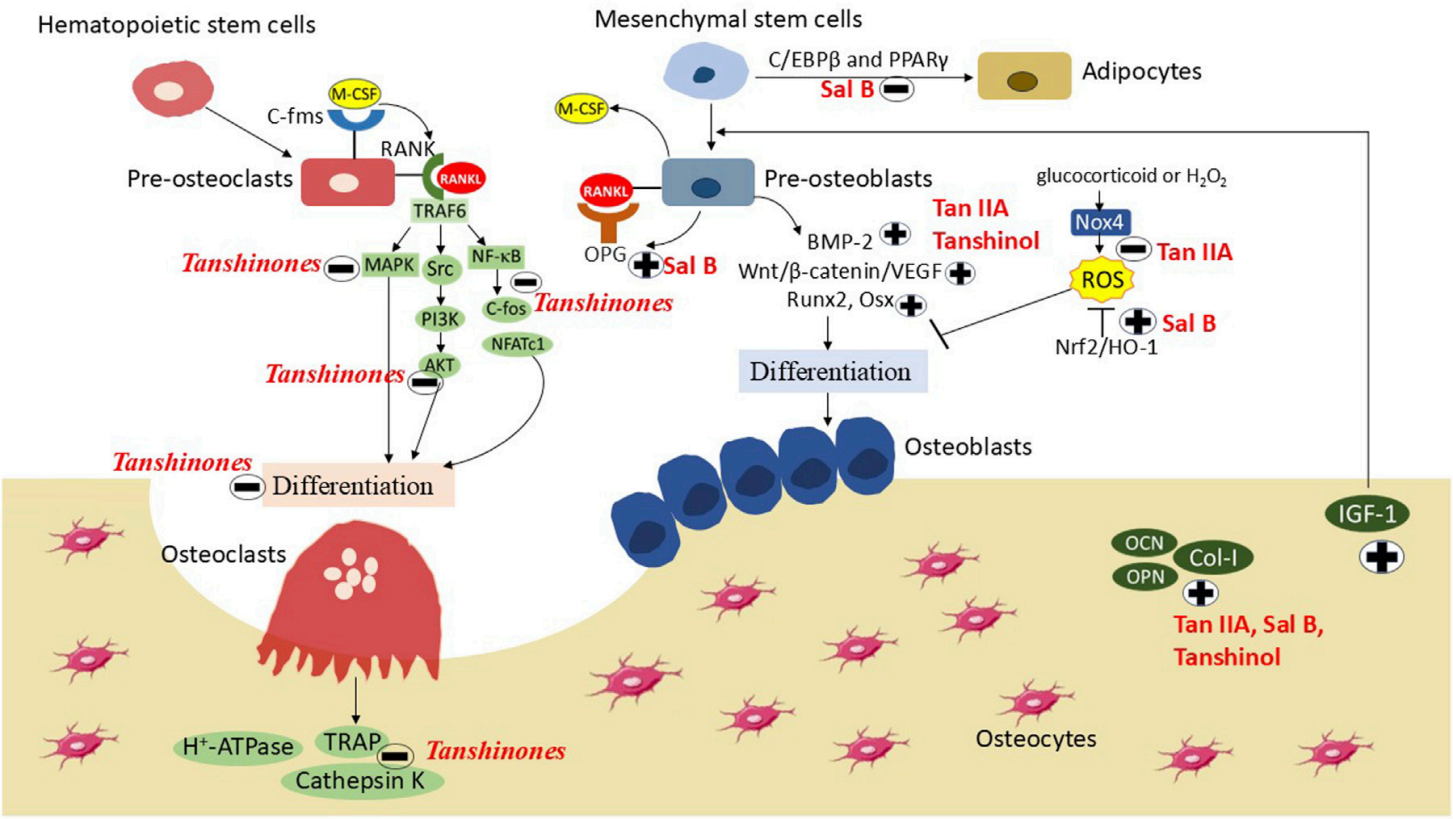
The actions and mechanisms of *Salvia Miltiorrhiza* on osteoporosis. Ethanol-soluble tanshinones inhibit RANKL (receptor activator of nuclear factor kappa-Β ligand) and M-CSF (macrophage colony-stimulating factor) induced osteoclasts differentiation and bone resorption via blocking the ERK (extracellular signal regulated kinase)/AKT (protein kinase B)/NF-κB (nuclear factor-kappa B) pathway. Both ethanol-soluble tanshinone IIA (Tan IIA) and water-soluble tanshinol and Sal B (salvianolic acid B) promote osteogenesis by limiting ROS (reactive oxygen species) over-production via regulating the Nox4 (NADPH oxidase 4)/Nrf2 (nuclear factor erythroid 2-related factor 2)/HO-1 (heme oxygenase 1) pathway, promoting osteogenesis and inhibiting adipogenesis via inhibiting the PPARγ (peroxisome proliferator-activated receptor γ) pathway and activating the Wnt (wingless-related integration site)/β-catenin and BMP (bone morphogenic protein)-2 pathways, as well as facilitating osteogenesis-angiogenesis coupling via the Wnt/β-catenin/VEGF (vascular endothelial growth factor) pathway.

SM has been historically and is currently either used alone or in combination with other herbs or non-plant components to treat skeletal diseases. Currently, more than 100 compounds have been identified from this herb. Of these, tanshinone IIA, cryptotanshinone, Sal B and tanshinol exert osteoprotective effects based on animal models and their corresponding cell models. The underlying mechanism may be involved in the inhibition of osteoclastogenesis/bone resorption by inhibiting the MAPK/NF-κB/NFATc1/cathepsin K signaling pathway, and promotion of osteoblastogenesis by promoting osteogenesis and angiogenesis via activation of the Wnt/β-catenin/VEGF and ERK/TAZ pathways, and eliminating ROS production targeting Nrf2/ARE/HO-1 pathway. However, recent interests only paid attentions to the effects of SM on osteoclasts and osteoblasts. Actions and metabolisms of SM on osteocytes should be considered in the future investigations, as osteocyte is one of the major bone cells and plays an important role in bone metabolism (Figure 4).

In the past 10 years, 38 clinical trials were documented that SM (single herbs or TCM prescriptions) could improve bone quality, attenuate osteoporosis pain and improve living quality in primary and secondary osteoporosis patients. However, the evidence is discounted due to the poor designed clinical trials and the limited duration of treatment. The criteria for efficacy were not clearly defined and unified, and biomarkers for the outcomes are diversified, thus making comparison and validation difficult. Therefore, randomized controlled trials with defined efficacy, a long-term duration, a large size of sample patients are needed in the future to evaluate the efficacy of SM on osteoporosis. In addition, human clinical trials validating the skeletal effects of tanshinones, Sal B and tanshinol are currently not available. For this purpose, rigorous clinical trials are warranted to substantiate the skeletal protective efficacy of these bioactive ingredients.

Combined with TCM theory, clinical usage and the results from preclinical investigations, SM may offer a new therapeutic avenue to treat osteoporosis and its complications. To this end, strong evidences from well-designed preclinical experiments and clinical trials are badly wanted, which will further enrich the applications of SM to prevent the development of osteoporosis.

## Glossary

AKT: protein kinase B
ALP or BALP: bone-specific alkaline phosphatase
αP2: adipocyte protein 2
ARE: anti-oxidant response element
BMC: bone mineral content
BMD: bone mineral density
BMP: bone morphogenic protein
BMMCs: bone marrow mononuclear cells
BMSCs: bone marrow mesenchymal stem cells
BGP: bone Gla protein or osteocalcin
BS/TV: bone surface/total tissue volume
BV/TV: bone volume/total tissue volume
C/EBPα: CCAAT-enhancer-binding protein α
Col-I: type I collagen
Conn.D: connectivity density
CTX-I: C-telopeptide cross-linked type I collagen
Dex: dexamethasone
**E**_**2**_: estradiol
ERK: extracellular signal regulated kinase
FoxO3a: forkhead box O3
GC: glucocorticoid
GSH-Px: glutathione peroxidase
HO-1: heme oxygenase 1
hMSCs: human mesenchymal stem cells
hPDLSCs: human periodontal ligament stem cells
IGF-I: insulin-like growth factor 1
KLF15: kruppel-like factor 15
LPS: lipopolysaccharide
M-CSF: macrophage colony-stimulating factor
MDA: malondialdehyde
MMP: matrix metalloproteinases
NFATc1: nuclear factor of activated T cells 1
NF-κB: nuclear factor-kappa B
NM: naturally menopaused
Nox4: NADPH oxidase 4
Nrf2: nuclear factor erythroid 2-related factor 2
OCN: osteocalcin
OPG: osteoprotegerin
OPN: osteopontin
Osx: Osterix
OVX: ovariectomized
PINP: procollagen type I N-propeptide
PPARγ: peroxisome proliferator-activated receptor γ
RANK: activator of nuclear factor kappa-Β ligand;
RANKL: receptor activator of nuclear factor kappa-Β ligand
Runx2: runt-related transcription factor 2
ROS: reactive oxygen species
Sal B: salvianolic acid B
SD: Sprague-Dawley
SM: Salvia Miltiorrhiza
SMG: simulated microgravity condition
SMI: structure model index
SOD: superoxide dismutase
STZ: streptozotocin/streptozocin
Tb.N: trabecular number
Tb.pf: trabecular pattern factor
Tb.Th: trabecular thickness
Tb.Sp: trabecular separation
TCM: traditional Chinese medicine
TRAF6: tumor necrosis factor receptor-associated factor 6
TRAP or TRACP: tartrate-resistant acid phosphatase
TXNIP: thioredoxin interacting protein
VAS: visual analogue scales
VEGF: vascular endothelial growth factor
Wnt: wingless-related integration site
25(OH)D: 25-hydroxyvitamin D
