# Supplementary material for: Salvia miltiorrhiza in osteoporosis: a review of its phytochemistry, traditional clinical uses and preclinical studies (2014–2024)
Source: Front Pharmacol. 2024 Oct 3;15:1483431. doi: 10.3389/fphar.2024.1483431 (PMC11484006; doi:10.3389/fphar.2024.1483431)
Supplement: Supplementary file 1 [file Table1.pdf]

Supplementary Table S1: Clinical osteoporosis trials containing *Salvia Miltiorrhiza*.

Study design	Efficacy of TCM (# of patients)<br>(VS before treatment)	Ref
**Prescription:** Danshen Injection<br>**Percentage of *SM*:** 100%<br>**Types of clinical trials:** Single center randomized controlled trial (RCT)<br>**Patients:** 86 cases of primary osteoporosis.<br>**Control (42 cases)**: Caltrate D for 6 months.<br>**TCM (44 cases)**: Danshen Injection combined with Caltrate D for 6 months.<br>**Measurements:** Lumbar BMD<br>**Clinical efficacy (Criteria ①):** Significant effect: complete disappearance of pain, significant increase in BMD; Effective: significant disappearance of pain, no decreases in BMD; Ineffective: no changes.	Lumbar BMD significantly increased by 0.029 g/cm^2, from 0.808 g/cm^2 to 0.837 g/cm^2.<br>**Clinical efficacy:**<br>Markedly improved (20)<br>Moderately improved (21)<br>Ineffective (3)<br>Overall efficacy:93.2%<br>Control treatment: 64.3%	(Chen et al., 2017)
**Prescription:** Tanshinone IIA sodium sulfonate injection<br>**Percentage of *SM*:** 100%<br>**Types of clinical trials:** Single center RCT<br>**Patients:** 60 cases of primary osteoporosis<br>**TCM 1 (30 cases)**: Sulfotanshinone Sodium Injection (80 mg/day) and Caltrate D for 2 weeks.<br>**TCM 2 (30 cases)**: TCM 1 combined acupuncture for 2 weeks.<br>**Measurements:** BMD; serum estradiol (E_2), BGP, PINP and β-CTX	**TCM 1:**<br>Lumbar BMD significantly increased by 0.007g/cm^2, from 0.793 g/cm^2 to 0.800 g/cm^2;<br>Femoral BMD significantly increased by 0.018 g/cm^2, from 0.764 g/cm^2 to 0.782 g/cm^2;<br>Significant increase in serum levels of PINP and E_2, and decrease in serum levels of BGP and β-CTX.<br>**TCM 2:**<br>Lumbar BMD significantly increased by 0.088g/cm^2(from 0.801 g/cm^2 to 0.889 g/cm^2);<br>Femoral BMD significantly increased by 0.099 g/cm^2 (from 0.753 g/cm^2 to 0.889 g/cm^2);<br>Significant increase in serum E_2 levels, and decrease in serum BGP levels.	(Chen et al., 2020)
**Prescription:** Tanshinone IIA sodium sulfonate injection<br>**Percentage of *SM*:** 100%<br>**Types of clinical trials:** Single center RCT<br>**Patients:** 80 cases of primary osteoporosis<br>**TCM 1 (40 cases)**: Sulfotanshinone Sodium Injection (60 mg/day by intravenous injection) and Caltrate D for 2 weeks.<br>**TCM 2 (40 cases)**: TCM 1 combined acupuncture for 2 weeks.<br>**Measurements:** PINP and β-CTX	**TCM 1:** no significant changes<br>**TCM 2:** Significant increase in serum PINP levels and decrease in β-CTX levels	(Yang et al., 2017)
**Prescription** [A]**:** Jianyao Migu Granule<br>**Percentage of *SM*:** 11.84%<br>**Types of clinical trials:** Double-blind multicenter randomized placebo-controlled trial<br>**Patients:** 99 cases of primary osteoporosis<br>**Control (49 cases)**: Jianyao Migu Granule placebo combined with Caltrate D (600 mg/day) for 6 months.<br>**TCM (50 cases)**: Jianyao Migu Granule combined with Caltrate	Lumbar BMD significantly increased by 0.14 g/cm^2 (from -1.54 g/cm^2 to -1.4 g/cm^2);<br>BMD of hip significantly increased by 0.10 g/cm^2(from -1.46 g/cm^2 to -1.36 g/cm^2);<br>Significant increase in serum TSH level and decrease in VAS pain score.	(Dai, 2020)

D (600 mg/day) for 6 months.<br>**Measurements:** BMD of lumbar and hip; serum Ca, P, PINP, β-CTX, 25(OH)D, ALP, BGP and TSH; VAS (Visual Analogue scales) pain score;		
**Prescription [B]:** Yiqi Bushen Huoxue Decoction<br>**Percentage of *SM*:** Not known<br>**Types of clinical trials:** Single center RCT<br>**Patients:** 60 cases of primary osteoporosis<br>**Control (30 cases)**: Caltrate D (1200 mg/day) for 3 months.<br>**TCM (30 cases)**: Yiqi Bushen Huoxue Decoction combined with Caltrate D (1200 mg/day) for 3 months.<br>**Measurements:** serum β-CTX, TRACP, PINP, and BGP.<br>**Criteria for clinical efficacy:** Recovered: disappearance of clinical symptoms; Significant effect: significant improvement in clinical symptoms; Effective: clinical symptoms alleviated; Ineffective: clinical symptoms remain the same.	Serum levels of BGP and PINP increased;<br>Serum levels of β-CTX and TRACP decreased.<br>**Clinical efficacy**<br>Recovered (9)<br>Markedly improved (11)<br>Moderately improved (8)<br>Ineffective (2)<br>Overall efficacy: 93.3%<br>Control treatment: 77.0%	(Wang et al., 2022)
**Prescription [C]:** Xianshen Bugu Yin<br>**Percentage of *SM*:** 20%<br>**Patients:** 50 cases of primary osteoporosis<br>**Types of clinical trials:** Single center RCT<br>**Control (25 cases)**: Calcitriol (0.5 μg/day) and Caltrate D for 3 months.<br>**TCM (25 cases)**: Xianshen Bugu Yin combined with western medicine (same as control) for 3 months.<br>**Measurements:** VAS score; serum levels of β-CTX and PINP.<br>**Criteria for clinical efficacy:** Significant effect: no significant pain in lumbar, recovery of spinal function; Effective: slight pain in lumbar, significant improvement of spinal function; Ineffective: no improvements.	Significant decrease in VAS score and serum levels of β-CTX;<br>Significant increase in serum levels of PINP.<br><br>**Clinical efficacy**<br>Markedly improved (11)<br>Moderately improved (12)<br>Ineffective (2)<br>Overall efficacy: 92%<br>Control treatment: 84%	(Bao, 2022)
**Prescription [D]:** Bushen Jiangu Decoction<br>**Percentage of *SM*:** 11.90%<br>**Types of clinical trials:** Single center RCT<br>**Patients:** 76 cases of primary osteoporosis<br>**Control (38 cases)**: Alendronate sodium (70 mg/week) for 3 months.<br>**TCM (38 cases)**: Bushen Jiangu Decoction combined with western medicine (same as control) for 3 months<br>**Measurements:** BMD of lumbar 2-4.<br>**Criteria for clinical efficacy:** Significant effect: significant improvement of clinical symptoms, TCM symptoms score decreased by ≥ 2/3, BMD increase ≥ 0.06 g/cm^2; Effective: improvement of clinical symptoms, TCM symptoms score decreased by 1/3~2/3, BMD increase < 0.06g/cm^2; Ineffective: no change of clinical symptoms, TCM symptoms score decreased by < 1/3, BMD remained the same or decreased.	BMD of lumbar 2 significantly increased by 0.22g/cm^2 (from 0.76 g/cm^2 to 0.98 g/cm^2);<br>BMD of lumbar 3 significantly increased by 0.23g/cm^2 (from 0.76 g/cm^2 to 0.99 g/cm^2);<br>BMD of lumbar 4 significantly increased by 0.3 g/cm^2 (from 0.76 g/cm^2 to 1.06 g/cm^2).<br>**Clinical efficacy**<br>Markedly improved (17)<br>Moderately improved (19)<br>Ineffective (2)<br>Overall efficacy: 94.74%<br>Control treatment: 71.05%	(Ru, 2016)
**Prescription [E]:** Shangke Yishen Zhuanggu Pill<br>**Percentage of *SM*:** Not known<br>**Types of clinical trials:** Single center RCT<br>**Patients:** 100 cases of primary osteoporosis	BMD of lumbar 2-4 significantly increased by 0.122 g/cm^2(from 0.774 g/cm^2 to 0.896 g/cm^2);<br>BMD of femoral neck significantly increased by 0.139 g/cm^2 (from 0.657 g/cm^2 to 0.796 g/cm^2);	(Mei, 2014)

**Control (50 cases)**: Caltrate D and salmon calcitonin injection (50 IU/day for 2 weeks and then 50 IU/week) for 6 months.<br>**TCM (50 cases)**: Shangke Yishen Zhuanggu Pill for 6 months.<br>**Measurements:** BMD of lumbar 2-4, femoral neck, Ward's triangle and trochanter; serum Ca, P, ALP; urine Ca, urine P; VAS pain score.<br>**Criteria for clinical efficacy:** Recovered: disappearance of clinical symptoms; score of pain and motion disability decreased by more than 2; Significant effect: significant improvement of clinical symptoms; score of pain and motion disability decrease >1; Effective: Improvement of clinical symptoms; Score of pain and motion disability remained the same as pretreatment; Ineffective: no improvements or even aggravated	BMD of Ward's triangle significantly increased by 0.174 g/cm^2 (from 0.479 g/cm^2 to 0.653 g/cm^2);<br>BMD of trochanter significantly increased by 0.121 g/cm^2 (from 0.535 g/cm^2 to 0.656 g/cm^2);<br>Significant increase in serum levels of ALP, Ca and decrease in serum P and urine Ca levels; Significant alleviation of pain.<br>**Clinical efficacy**<br>Recovered (2)<br>Markedly improved (15)<br>Moderately improved (30)<br>Ineffective (3)<br>Overall efficacy: 94%<br>Control treatment: 76%	
**Prescription [F]:** Young Bushen Huoxue Soup<br>**Percentage of *SM*:** 8.70%<br>**Types of clinical trials:** Single center RCT<br>**Patients:** 50 cases of primary osteoporosis<br>**Control (25 cases):** Caltrate D (1200 mg/day) and celecoxib (200 mg/day) for 24 weeks<br>**TCM (25 cases)**: Bushen Jiangu Decoction combined with western medicine (same as control) for 24 weeks<br>**Measurements:** BMD, serum level of Ca.<br>**Criteria for clinical efficacy:** Significant effect: Pain decreased by more than 4 grades, or at least 2 laboratory indexes increased, or BMD increased; Effective: pain decreased by more than 2 grades, or no significant changes in laboratory indicators, or no decreases in BMD; Ineffective: no improvement of pain, a decrease in at least 2 laboratory measures such as serum Ca, or a continued decrease in BMD.<br>**Criteria ② of TCM efficacy:** Cured: disappearance of symptoms, N ≥ 95%; Significant effect: significant improvement of symptoms, N ≥ 70%; Effective: improvement of symptoms, N ≥ 30%; Ineffective: symptoms remained the same or even aggravated, N < 30%.<br>N = (pre-therapy symptoms score - post-therapy symptoms score)/ pre-therapy symptoms score	BMD significantly increased by 0.12 g/cm^2 (from 0.54 g/cm^2 to 0.66 g/cm^2);<br>Significant increase in serum Ca levels.<br>**Clinical efficacy:**<br>Markedly improved (15)<br>Moderately improved (9)<br>Ineffective (1)<br>Overall efficacy: 96%<br>Control treatment: 60%<br>**TCM efficacy:**<br>Cured (1)<br>Markedly improved (11)<br>Moderately improved (11)<br>Ineffective (2)<br>Overall efficacy: 92%<br>Control treatment: 64%	(Pan, 2014)
**Prescription [G]:** Bushen Huoxue Decoction<br>**Percentage of *SM*:** 6.25%<br>**Types of clinical trials:** Single center RCT<br>**Patients:** 66 cases of primary osteoporosis<br>**Control (33 cases)**: Caltrate D, calcitriol and salmon calcitonin Nasal Spray (360 IU/day at first week and then 360 IU/2 day) for 4 weeks.<br>**TCM (33 cases)**: Bushen Huoxue Decoction combined with western medicine (same as control) for 4 weeks.<br>**Measurements:** BMD of lumbar 2-4 and hips; VAS pain score,<br>**Criteria ③ for clinical efficacy:**<br>Cured: Complete disappearance of symptoms such as pain,	Lumbar BMD increased by 0.3 g/cm^2(from -2.96 g/cm^2 to -2.66 g/cm^2);<br>BMD of left hip increased by 0.12 g/cm^2 (from -2.62 g/cm^2 to -2.50 g/cm^2);<br>Significant alleviation of pain.<br><br>**Clinical efficacy:**<br>Cured (5)<br>Markedly improved (14)<br>Moderately improved (8)<br>Ineffective (1)<br>Overall efficacy: 96.43%	(Dai, 2022)

motion disability, and tinnitus, N > 95%; Significant effect: significant improvement of symptoms, N between 70%~95%; Effective: improvement of symptoms, N between 30%~69%; Ineffective: symptoms remain the same or even aggravation, N <30%.		
**Prescription [H]:** Bushen Jiangu Prescription<br>**Percentage of *SM*:** 9.375%<br>**Types of clinical trials:** Single center RCT<br>**Patients:** 30 cases of primary osteoporosis<br>**Control (15 cases):** no treatment.<br>**TCM 1 (15 cases):** Gu Kang capsule for 8 weeks.<br>**TCM 2 (15 cases)**: Bushen Jiangu Prescription for 8 weeks.<br>**Criteria for clinical efficacy:** same as above **Criteria ③**	**Clinical efficacy:**<br>Cured (1)<br>Markedly improved (8)<br>Moderately improved (5)<br>Ineffective (1)<br>Overall efficacy: 93.33%<br>Control treatment: 73.33%	(Yang, 2015)
**Prescription [I]:** Gujian Decoction<br>**Percentage of *SM*:** 9%<br>**Types of clinical trials:** Single center RCT<br>**Patients:**100 cases of primary osteoporosis<br>**Control (50 cases)**: Calcitriol (0.5 μg/day) and caltrate D calcium carbonate D3 tablets (calcium carbonate 1.25 g/day, vitamin D3 200 IU/ day) for 3 months.<br>**TCM (50 cases)**: Gujian Decoction combined with western medicine (same as control) for 3 months.<br>**Measurements:** Lumbar biomechanics<br>**Criteria for TCM efficacy:** same as above **Criteria ③**	Significant increase in lumbar biomechanical strength.<br><br>**Clinical efficacy:**<br>Markedly improved (10)<br>Moderately improved (37)<br>Ineffective (3)<br>Overall efficacy: 94%<br>Control treatment: 76%	(Chu, 2019)
**Prescription [I]:** Gujian Decoction<br>**Percentage of *SM*:** 9 %<br>**Types of clinical trials:** Single center RCT<br>**Patients:**100 cases of primary osteoporosis<br>**Control (50 cases)**: Calcitriol (0.5 μg/day) and caltrate D (Calcium carbonate 1.5 g/day) for 3 months.<br>**TCM (50 cases)**: Gujian Decoction, combined with western medicine (same as control) for 3 months.<br>**Measurements:** CTX, Ca, P in serum;<br>**Criteria for clinical efficacy:** Significant effect: 70% ≥ N < 100%; Effective: 30% ≤N <70%; Ineffective: N ≤ 30%. (N same as above)	Significant decrease in serum levels of CTX.<br>**Clinical efficacy:**<br>Markedly improved (21)<br>Moderately improved (26)<br>Ineffective (3)<br>Overall efficacy: 94%<br>Control treatment: 76%	(Zheng, 2018)
**Prescription [I]:** Gujian Decoction<br>**Percentage of *SM*:** 9 %<br>**Types of clinical trials:** Single center RCT<br>**Patients:** 78 cases of postmenopausal osteoporosis<br>**Control (39 cases)**: Jin Tian Ge capsule (3.6 g/day) for 3 months.<br>**TCM (39 cases)**: Gujian Decoction for 3 months.<br>**Measurements:** Balance test; Serum E_2.<br>**Criteria for clinical efficacy:** Significant effect: N > 60%; Effective: 25% ≤ N ≤ 60%; Ineffective: N < 25%. (N same as above)	Significant increase in serum E_2 levels, lumbar function and balance ability.<br>**Clinical efficacy:**<br>Markedly improved (17)<br>Moderately improved (19)<br>Ineffective (3)<br>Overall efficacy: 92.31%<br>Control treatment: 82.05%	(Chen, 2020)
**Prescription [L]:** Jianyao Migu Prescription<br>**Percentage of *SM*:**13.64% of SM<br>**Types of clinical trials:** Single center RCT	Significant increase in BMD (from -2.95 g/cm^2 to -2.94 g/cm^2);<br>Significant increase in serum levels of Ca, PINP and	(He, 2019)

**Patients:** 100 cases of postmenopausal osteoporosis<br>**Control (50 cases)**: Calcitriol (0.25 μg/day) for 6 months.<br>**TCM (50 cases)**: Jianyao Migu Prescription combined with calcitriol for 6 months.<br>**Measurements:** BMD of calcaneus; Serum Ca, P, ALP, PINP, β-CTX, 25(OH)D and PTH.<br>**Criteria for clinical efficacy:** same as above **Criteria ③**	25(OH)D;<br>Significant decrease in serum levels of β-CTX and PTH;<br>**Clinical efficacy:**<br>Markedly improved (40)<br>Moderately improved (4)<br>Ineffective (6)<br>Overall efficacy: 88%<br>Control treatment: 76%	
**Prescription** [K]**:** JiaWeiErXian Decoction<br>**Percentage of *SM*:** 8%<br>**Types of clinical trials:** Single center RCT<br>**Patients:** 60 cases of postmenopausal osteoporosis<br>**Control (30 cases)**: Caltrate D (600 mg/day) for 12 weeks.<br>**TCM (30 cases)**: JiaWeiErXian Decoction combined with calcitriol for 12 weeks.<br>**Measurements:** BMD of lumbar 2-4; serum Ca, P, OCN, β-CTX, 25(OH)D; VAS score.<br>**Criteria for clinical efficacy:** same as above **Criteria ①**<br>**Criteria for TCM efficacy:** same as above **Criteria ③**	Significant increase in BMD (from -2.95 g/cm^2 to -2.91 g/cm^2);<br>Significant decrease in VAS pain scores and increase in serum levels of 25(OH)D.<br>**Clinical efficacy:**<br>Markedly improved (4)<br>Moderately improved (22)<br>Ineffective (4)<br>Overall efficacy: 86.7%<br>Control treatment: 66.7%<br>**TCM efficacy:**<br>Markedly improved (7);<br>Moderately improved (22)<br>Ineffective (1)<br>Overall efficacy: 96.7%<br>Control treatment: 86.7%	(Zhang, 2019)
**Prescription** [L]**:** Bushen Huoxue Decoction<br>**Percentage of *SM*:** Not known<br>**Types of clinical trials:** Single center RCT<br>**Patients:** 80 cases of postmenopausal primary osteoporosis<br>**Control (38 cases)**: 600 mg/day caltrate D for 90 days.<br>**TCM (42 cases)**: Bushen Huoxue Decoction combined with calcitriol for 90 days.<br>**Measurements:** BMD of lumbar 1-4, femur, Troch, Ward's; Serum levels of BGP, ALP, Deoxypyridinoline (DPD), TRACP5b.<br>**Criteria for clinical efficacy:** Cured: disappearance of symptoms such as pain, motion disability, and tinnitus, N > 90%; Significant effect: significant improvement of symptoms, N between 70%~90%; Effective: improvement of symptoms, N between 30%~70%; Ineffective: symptoms remain the same or even aggravation, N < 30%. (N same as above)	Lumbar BMD significantly increased by 0.12 g/cm^2,(from 0.75 g/cm^2 to 0.87 g/cm^2);<br>Ward's BMD significantly increased by 0.15 g/cm^2 (from 0.42 g/cm^2 to 0.57 g/cm^2);<br>Marked improvement of pain;<br>Significant decrease in serum levels of BGP, ALP, DPD and TRACP5b.<br>**Clinical efficacy:**<br>Cured (9)<br>Markedly improved (11)<br>Moderately improved (18)<br>Ineffective (4)<br>Overall efficacy: 90.48%<br>Control treatment: 76.32%	(Han, 2016)
**Prescription** [M]**:** Yiyin Zhuanggu Pill<br>**Percentage of *SM*:** 8%<br>**Types of clinical trials:** Single center RCT<br>**Patients:** 72 cases of postmenopausal osteoporosis<br>**Control (36 cases)**: Alendronate (70 mg/week) combined with caltrate D (including Ca 600 mg/day, vitamin D3 125 IU/day) and calcitriol (0.25 μg/day) for 24 weeks.<br>**TCM (36 cases)**: Yiyin Zhuanggu Pill combined with caltrate D and calcitriol (same as control) for 24 weeks.<br>**Measurements:** BMD of hip	BMD of hip significantly increased by 0.107 g/cm^2 (from 0.635 g/cm^2 to 0.742 g/cm^2).<br><br>**Clinical efficacy:**<br>Markedly improved (9)<br>Moderately improved (19)<br>Ineffective (4)<br>Overall efficacy: 87.50%<br>Control treatment: 67.65%	(Li, 2021)

**Criteria ④ for clinical efficacy:** Significant effect: clinical symptoms and signs significantly improved; N ≥ 70%; Effective: clinical symptoms and signs improved; 30% ≤ N < 70%; Ineffective: clinical symptoms and signs remain the same, N < 30%. (N same as above)		
**Prescription [N]:** ZhuangguFang Decoction<br>**Percentage of *SM*:** 14.29%<br>**Types of clinical trials:** not known<br>**Patients:** 90 cases of postmenopausal osteoporosis<br>**Control (30 cases)**: Caltrate D (1.25 g/day) and calcitriol (0.25 μg/day) for 6 months.<br>**TCM 1 (30 cases)**: ZhuangguFang Decoction for 6 months.<br>**TCM 2 (30 cases)**: Zhuanggu Fang Decoction combined with western medicine (same as control) for 6 months.<br>**Measurements:** Lumbar hydroxyapatite (QCT BMD); 25(OH) D, OCN, PINP, β-CTX in serum; TCM symptoms score.	**TCM 1:**<br>Significant decrease in serum levels of OCN and β-CTX.<br>**TCM 2:**<br>Significant increase in lumbar hydroxyapatite (QCT BMD) and serum levels of 25 (OH)D;<br>Significant decrease in serum levels of OCN and β-CTX.	(Xie, 2018)
**Prescription [O]:** Blood Repellent Bruises<br>**Percentage of *SM*:** 5.88%<br>**Types of clinical trials:** Single center RCT<br>**Patients:** 88 cases of postmenopausal osteoporosis<br>**Control (44 cases)**: Calcium carbonate D3 tablets (500 mg/day), calcitriol (every day) and zoledronic acid (once a year) for 1 year.<br>**TCM (44 cases)**: Blood Repellent Bruises combined with western medicine (same as control) for 1 year.<br>**Measurements:** BMD of femoral troch, ward's and lumbar 1-4;<br>**Criteria of efficacy:** Significant effect: disappearance of clinical symptoms, BMD increase by > 10%; Effective: clinical symptoms relieve, BMD increase by ≤ 10%; Ineffective: on change of clinical symptoms, BMD increase by ≤ 10%; Aggravated: clinical symptoms aggravated, BMD remained the same or even decreased.	BMD of femoral troch markedly increased by 0.55 g/cm^2 (from -2.76 g/cm^2 to -2.01 g/cm^2);<br>BMD of Ward's markedly increased by 0.72 g/cm^2 (from -2.84 g/cm^2 to -2.12 g/cm^2);<br>BMD of lumbar 1-4 markedly increased by 0.91 g/cm^2(from -2.9 g/cm^2 to -1.99 g/cm^2).<br>**Clinical efficacy:**<br>Markedly improved (10)<br>Moderately improved (24)<br>Ineffective (9)<br>Aggravated (1)<br>Overall efficacy: 77%<br>Control treatment: 54%	(Hong, 2022)
**Prescription [P]:** Aolong Granule<br>**Percentage of *SM*:** 5%<br>**Types of clinical trials:** not known<br>**Patients:** 60 cases of perimenopausal osteoporosis<br>**Control (30 cases)**: Xianling Gubao Capsule for 30 days.<br>**TCM (30 cases)**: Aolong Granule for 30 days.<br>**Measurements:** BMD<br>**Efficacy evaluation:** Significant effect: pain decreased from grade III or II to grade 0; Effective: pain decreased from grade III or II to grade I; Ineffective: pain remained the original levels or above grade II.<br>VAS pain score: grade 0: 0 score, no pain; grade I: < 3 score, slight pain, can endure; grade II: 4-6 score, pain affect sleep but can endure; grade III: 7-10 score, severe pain and unbearable	BMD significantly increased by 1.27 g/cm^2 (from 0.74 g/cm^2 to 2.01 g/cm^2).<br><br>**Clinical efficacy:**<br>Markedly improved (14)<br>Moderately improved (12)<br>Ineffective (4)<br>Overall efficacy: 86.67%<br>Control treatment: 83.33%	(Liu et al., 2015)
**Prescription [Q]:** GuShuBao Recipe<br>**Percentage of *SM*:** Not known<br>**Types of clinical trials:** Single center RCT<br>**Patients:** 52 cases of perimenopausal osteoporosis	BMD of lumbar 1-4 significantly increased by 0.051 g/cm^2 (from 0.686 g/cm^2 to 0.737 g/cm^2);<br>BMD of left hip increased by 0.045 g/cm^2 (from 0.715 g/cm^2 to 0.760 g/cm^2);	(Kong, 2014)

**Control (26 cases)**: Xianling Gubao Capsule (3 g/day) for 6 months.<br>**TCM (26 cases)**: GuShuBao Recipe for 6 months.<br>**Measurements:** BMD; serum ALP, E_2, follicle-stimulating hormone (FSH) and Ca;<br>**Criteria for clinical efficacy:** Significant effect: disappearance of pain in back and loin; Pain scores decreased by ≥ 2/3, BMD of lumbar spine and left femoral neck significantly increased by 0.05 g/cm^2; Effective: obvious improvement of back and loin pain, pain scores decreased by ≥ 2/3, BMD significantly increased but less than 0.05 g/cm^2; Ineffective: pain and other symptoms remained the same, pain scores decreased by ≤1/3, BMD did not change or even decreased.<br>**Criteria for TCM efficacy:** same as above **Criteria ③**	Significant increase in serum levels of E_2 and Ca, and decrease in serum levels of FSH and ALP.<br>**Clinical efficacy:**<br>Markedly improved (10)<br>Moderately improved (13)<br>Ineffective (3)<br>Overall efficacy: 88%<br>Control treatment: 62%<br>**TCM efficacy:**<br>Markedly improved (12)<br>Moderately improved (11)<br>Ineffective (3)<br>Overall efficacy: 88.5%<br>Control treatment: 61.5%	
**Prescription** [R]: Bushen Huoxue Recipe<br>**Percentage of *SM*:** 10.75%<br>**Types of clinical trials:** Single center RCT<br>**Patients**: 120 cases of senile osteoporosis<br>**Control (60 cases)**: Caltrate D (1200 mg/day calcium and vitamin D3 250 IU/day) for 3 months.<br>**TCM (60 cases)**: Bushen Huoxue Recipe combined with caltrate D (same as control) for 3 months<br>**Measurements:** BMD, serum E_2<br>**NRS score:** Grade 0: no pain; grade 1 to 3: mild pain; grade 4 to 6: moderate pain; grade 7 to 10: severe pain.	BMD significantly increased by 0.38 g/cm^2 (from -2.63 g/cm^2 to -2.25 g/cm^2);<br>Significant increase in serum E_2 levels and decrease in NRS scores.	(Jiang, 2015)
**Prescription** R: Bushen Huoxue Recipe<br>**Percentage of *SM*:** 11.15%<br>**Types of clinical trials:** Single center RCT<br>**Patients**: 200 cases of senile osteoporosis<br>**Control (100 cases)**: Caltrate D for 3 months.<br>**TCM (100 cases)**: Bushen Huoxue Recipe combined with caltrate D for 3 months.<br>**Measurements:** BMD of lumbar 2-4 and femoral troch; serum BGP, E_2; NRS score (same as above)	Lumbar BMD (0.958 g/cm^2) significantly higher than that of control (0.784 g/cm^2);<br>Femoral troch BMD (0.916 g/cm^2) significantly higher than that of control (0.761 g/cm^2);<br>Serum E_2 levels increased;<br>Serum BGP levels decreased.	(Chai et al., 2015)
**Prescription** [S]**:** Salvia and Rehmannia for Relieving Pain Formula<br>**Percentage of *SM*:** 7.41%<br>**Types of clinical trials:** Single center RCT<br>**Patients:** 70 cases of senile osteoporosis<br>**Control (35 cases)**: Caltrate D (600 mg/day), and salmon calcitonin (50 IU/day at first 2 weeks and then 50 IU/2 weeks by intramuscular injection) for 3 months.<br>**TCM (35 cases)**: Salvia and Rehmannia for Relieving Pain Formula combined with western medicine (same as control) for 3 months.<br>**Measurements:** BMD of lumbar 2-4, femoral neck and femoral troch; VAS pain score; Oswestry disability index (ODI); activity of daily living (ADL)<br>**Criteria for clinical efficacy:** Significant effect: significant	BMD of lumbar 2-4 significantly increased by 11.18 mg/cm^2 (from 61.04 g/cm^2 to 72.22 g/cm^2);<br>BMD of femoral neck significantly increased by 12.63 mg/cm^2(from 65.52 g/cm^2 to 78.15 g/cm^2);<br>BMD of femoral troch significantly increased by 10.68 mg/cm^2;<br>Significant decrease in VAS scores and ODI, and increase in ADL.<br><br>**Clinical efficacy:**<br>Markedly improved (22)<br>Moderately improved (11)<br>Ineffective (2)<br>Overall efficacy: 94.29%<br>Control treatment: 77.14%	(Wang, 2022)

increase of BMD, disappearance of clinical symptoms; Effective: most of clinical symptoms disappeared; Occasional pain but not significantly affected normal life; Ineffective: No improvement in biomarkers.		
**Prescription [T]:** DanShen HuangQi Decoction<br>**Percentage of *SM*:** 7.69%<br>**Types of clinical trials:** Single center RCT<br>**Patients:** 82 cases of senile osteoporosis<br>**Control (41 cases)**: Caltrate D (600 mg/day), and salmon calcitonin (50 IU/day at first 2 weeks and then 50 IU/2 times/2 weeks by intramuscular injection) for 3 months.<br>**TCM (41 cases)**: DanShen HuangQi Decoction combined with western medicine (same as control) for 3 months.<br>**Measurements:** BMD of lumbar; serum levels of β-CTX, PINP, BGP; VAS pain score;<br>**Criteria for clinical efficacy:** Significant effect: disappearance of back and bone pain, significant increase of BMD; Effective: occasional back pain but did not affect normal life; BMD improved; Ineffective: no improvement or even aggravate.	Lumbar BMD: significantly increased by 10.61 mg/cm^2 (from 59.84 mg/cm^2 to 70.45 mg/cm^2);<br>Significant decrease in VAS score and serum levels of β-CTX;<br>Significant increase in serum BGP levels.<br><br>**Clinical efficacy:**<br>Markedly improved (17)<br>Moderately improved (20)<br>Ineffective (4)<br>Overall efficacy: 90. 24%<br>Control treatment: 70. 73%	(Su et al., 2020)
**Prescription [U]:** Yiqi Huoxue Zhuanggu Formula<br>**Percentage of *SM*:** 14.29%<br>**Types of clinical trials:** Single center RCT<br>**Patients:** 180 cases of senile osteoporosis with coronary disease<br>**Control (90 cases)**: Caltrate D, calcitriol and alendronate sodium for 3 months.<br>**TCM (90 cases)**: Yiqi Huoxue Zhuanggu Formula combined with western medicine (same as control) for 3 months.<br>**Measurements:** Serum CTX-1 and BGP, BMD of lumbar 1-4.<br>**Criteria for clinical efficacy:** Significant effect: lumbar 1-4 BMD increased by more than 20%; Effective: BMD increased by more than 10% but less than 20%; Ineffective: did not meet the above criteria.	BMD of lumbar 1-4 significantly increased by 0.43 g/cm^2 (from 0.61 g/cm^2 to 1.04 g/cm^2);<br>Significant decrease in serum CTX-1 levels and significant increase in serum BGP levels.<br>**Clinical efficacy:**<br>Markedly improved (43)<br>Moderately improved (39)<br>Ineffective (8)<br>Overall efficacy: 91.11%<br>Control treatment: 80.00%	(Cai and Deng, 2015)
**Prescription [V]:** Yishen Qianggu Mixture<br>**Percentage of *SM*:** 23.8%<br>**Types of clinical trials:** Single center RCT<br>**Patients:** 180 cases of senile osteoporosis<br>**Control (90 cases)**: 1200 mg/day caltrate D for 12 months.<br>**TCM (90 cases)**: Yishen Qianggu Mixture combined with caltrate D (same as control) for 12 months.<br>**Measurements:** BMD of hip and lumbar; serum BGP, calcitonin, β-Crossla, PINP<br>**Criteria for TCM efficacy:** same as above **Criteria ④**	Lumbar BMD markedly increased by 0.26 g/cm^2 (from -2.55 g/cm^2 to -2.29 g/cm^2);<br>Hip BMD markedly increased by 0.16 g/cm^2(from -1.15 g/cm^2 to -0.99 g/cm^2);<br>Significant increase in serum levels of BGP, calcitonin and T-PINP;<br>Significant decrease in serum levels of β-Crossla.<br>**Clinical efficacy:**<br>Markedly improved (66)<br>Moderately improved (21)<br>Ineffective (3)<br>Overall efficacy: 96.67%<br>Control treatment: 77.78%	(Cao et al., 2021)
**Prescription [W]:** Yishen Jiangu Prescription<br>**Percentage of *SM*:** 10.27%<br>**Types of clinical trials:** Single center RCT<br>**Patients:** 60 cases of diabetic osteoporosis	BMD of femoral neck increased by 0.02 g/cm^2 (from 0.55 g/cm^2 to 0.57 g/cm^2); BMD of lumbar increased by 0.01 g/cm^2 (from 0.74 g/cm^2 to 0.75 g/cm^2);<br>Significant increase in serum levels of PINP and	(Shi, 2018)

**Control (30 cases)**: Calcium and vitamin D for 3 months.<br>**TCM (30 cases)**: Yishen Jiangu Prescription combined with basic supplement (same as control) for 3 months.<br>**Measurements:** Femur and lumbar BMD; serum PINP, s-CTX, 25(OH)D, PTH, Ca and P; urine Ca and P.<br>**Criteria for TCM efficacy:** same as above **Criteria** ④	25(OH)D;<br>Significant decrease in serum CTX levels and urine Ca levels.<br>**Clinical efficacy:**<br>Markedly improved (6)<br>Moderately improved (19)<br>Ineffective (5)<br>Overall efficacy: 83.3%<br>Control treatment: 50%	
**Prescription [X]:** Bushen Huoxue Decoction<br>**Percentage of *SM*:** 15.08%<br>**Types of clinical trials:** Single center RCT<br>**Patients:** 70 cases of diabetic osteoporosis<br>**Control (34 cases)**: Bone strengthening capsules for 8 weeks.<br>**TCM (36 cases)**: Bushen Huoxue Decoction for 8 weeks.<br>**Measurements:** β-CTX, OCN, PINP, 25(OH)D in serum.<br>**Criteria for TCM efficacy:** same as above **Criteria** ②	Significant decrease in serum β-CTX levels;<br>Significant increase in serum levels of PINP, 25(OH)D and OCN.<br>**TCM efficacy:**<br>Markedly improved (19)<br>Moderately improved (14)<br>Ineffective (3)<br>Overall efficacy: 91.7%<br>Control treatment: 47.1%	(Fu, 2021)
**Prescription [Y]:** GuShuKang Capsule<br>**Percentage of *SM*:** Not known<br>**Types of clinical trials:** Single center RCT<br>**Patients:** 105 cases of diabetic osteoporosis<br>**Control 1 (35 cases)**: Rocalirol (0.25 μg/day) and caltrate D (600 mg/day) for 6 months.<br>**Control 2 (35 cases)**: Rocalirol (0.25 μg/day), caltrate D (600 mg/day) and alendronate sodium (70 mg/week) for 6 months.<br>**TCM (35 cases)**: GuShuKang Capsule, rocalirol (0.25 μg/day) and caltrate D (600 mg/day) for 6 months.<br>**Measurements:** BMD of lumbar 1-4 and femur; Serum levels of Ca, P, ALP, PINP, β-CTX, BGP and vitamin D.<br>**Criteria for clinical efficacy:** Significant effect: disappearance of symptoms, BMD recovered to normal; no abnormality in bone metabolism indicators or recover > 40%; Effective: significant improvement of symptoms, marked improvement of BMD and bone metabolism indicators; Ineffective: no changes.<br>**Criteria for TCM efficacy:** same as above **Criteria** ②	BMD of lumbar 1-4 significant increased by 0.08 g/cm^2(from 0.70 g/cm^2 to 0.78 g/cm^2);<br>BMD of femoral neck significantly increased by 0.55 g/cm^2(from 0.60 g/cm^2 to 1.15 g/cm^2);<br>Significant increase in serum levels of PINP, BGP, Ca and vitamin D;<br>Significant decrease in serum levels of P and β-CTX.<br>**Clinical efficacy:**<br>Markedly improved (14)<br>Moderately improved (18)<br>Ineffective (3)<br>Overall efficacy: 91.4%<br>Control 1 treatment: 54.2%<br>Control 2 treatment: 82.8%	(Shuai, 2018)
**Prescription [Z]:** Yiqi Tongluo Prescription<br>**Percentage of *SM*:** 7.23%<br>**Types of clinical trials:** Single center RCT<br>**Patients:** 60 cases of diabetic osteoporosis<br>**Control (30 cases)**: Caltrate D (1200 mg/day) for 6 months<br>**TCM (30 cases)**: Yiqi Tongluo Prescription and caltrate D (same as control) for 6 months<br>**Measurements:** Lumar BMD; Serum Ca, P, ALP, OCN, PINP, E_2 and PTH<br>**Criteria for clinical efficacy:** Same as above **Criteria** ①<br>**Criteria for TCM efficacy:** Same as above **Criteria** ②	Lumbar BMD significantly increased by 0.042 g/cm^2 (from 0.701 g/cm^2 to 0.743 g/cm^2)<br>Significant increase in serum levels of Ca, OCN, PINP, and E_2;<br>Significant decrease in serum PTH levels.<br>**Clinical efficacy:**<br>Markedly improved (8)<br>Moderately improved (18)<br>Ineffective (4)<br>Overall efficacy: 86.67%<br>Control treatment: 63.33%	(Wang, 2018)
**Prescription [AA]:** Guben Huoxue prescription<br>**Percentage of *SM*:** 7.25%	Male lumbar BMD significantly increased by 0.046 g/cm^2(from 0.810 g/cm^2 to 0.856 g/cm^2);	(Li, 2018)

**Types of clinical trials:** Single center RCT<br>**Patients:** 60 cases of diabetic osteoporosis<br>**Control (30 cases)**: Caltrate D (600 mg/day) and alfacalcidol (0.5 ug/day) for 6 months<br>**TCM (30 cases)**: Guben Huoxue prescription combined with western medicine (same as control) for 6 months<br>**Measurements:** BMD of lumbar 1-4; Serum Ca, P, ALP, BGP, PTH, vitamin D, PINP, β-CTX, E_2 and testosterone.<br>**Criteria for clinical efficacy:** Same as above **Criteria ①**	Female lumbar BMD significantly increased by 0.066 g/cm^2(from 0.745 g/cm^2 to 0.811 g/cm^2);<br>Significant increase in serum levels of BGP, Ca, ALP, PINP, and vitamin D;<br>Significant decrease in serum levels of P, PTH, and β-CTX.<br>**Clinical efficacy**<br>Markedly improved (8)<br>Moderately improved (20)<br>Ineffective (2)<br>Overall efficacy:93.3%<br>Control treatment: 70.0%	
**Prescription [BB]:** Jianpi Yishen Huoxue Recipe<br>**Percentage of *SM*:** Not known<br>**Types of clinical trials:** Single center RCT<br>**Patients:** 60 cases of diabetic osteoporosis<br>**Control** (30 cases): alendronate sodium (70 mg/week) for 6 months.<br>**TCM** (30 cases): Jianpi Yishen Huoxue Recipe and alendronate sodium (same as control) for 6 months<br>**Measurements:** BMD of lumbar 1-4, femoral neck, Torch and Ward's; serum levels of Ca, P, PTH, PINP and β-CTX.<br>**Criteria for clinical efficacy:** Significant effect: disappearance of osteoporosis pain, increase of BMD; Effective: pain significantly alleviated, no decrease of BMD; Ineffective: clinical symptoms remain the same or even aggravated	BMD of lumbar 1 significantly increased by 0.088 g/cm^2 (from 0.750 g/cm^2 to 0.838 g/cm^2);<br>BMD of lumbar 2 significantly increased by 0.059 g/cm^2 (from 0.800 g/cm^2 to 0.859 g/cm^2);<br>BMD of lumbar 3 significantly increased by 0.067 g/cm^2(from 0.741 g/cm^2 to 0.808 g/cm^2);<br>BMD of lumbar 4 significantly increased by 0.077 g/cm^2 (from 0.789 g/cm^2 to 0.866 g/cm^2);<br>BMD of femoral neck significantly increased by 0.061 g/cm^2 (from 0.771 g/cm^2 to 0.832 g/cm^2);<br>BMD of torch significantly increased by 0.060 g/cm^2 (from 0.840 g/cm^2 to 0.900 g/cm^2);<br>BMD of Ward's: significantly increased by 0.047 g/cm^2(from 0.803 g/cm^2 to 0.850 g/cm^2);<br>Significant increase in serum Ca levels and decrease in serum levels of P, PTH, PINP and β-CTX.<br>**Clinical efficacy:**<br>Markedly improved (12)<br>Moderately improved (14)<br>Ineffective (4)<br>Overall efficacy: 86.7%<br>Control treatment: 63.3%	(Xu, 2020)
**Prescription [CC]:** Zuogui Pill and HuoLuo XiaoLing Dan<br>**Percentage of *SM*:** 9.09%<br>**Types of clinical trials:** Single center RCT<br>**Patients:** 70 cases of osteoporosis<br>**Control (35 cases):** Caltrate D (600 mg/day Ca, 125 IU/day vitamin D) and alendronate sodium (70 mg/week) for 1 month.<br>**TCM (35 cases):** Zuogui Pill and HuoLuo XiaoLing Dan combined with western medicine (same as control) for 1 month.<br>**Measurements:** VAS pain score<br>**Criteria for clinical efficacy:** Same as above **Criteria ③**	Significant decrease in VAS pain scores.<br>**Clinical efficacy:**<br>Cured (0)<br>Significant effect (22)<br>Effective (8)<br>Ineffective (5)<br>Overall efficacy: 85.71%<br>Control treatment: 60%	(Gong, 2019)
**Prescription [DD]:** Yigu Decoction<br>**Percentage of *SM*:** 30%<br>**Types of clinical trials:** Single center RCT<br>**Patients:** 70 cases of osteoporosis<br>**Control (35 cases)**: Vitamin D (400 U/day) and calcium	BMD increased by 0.022 g/cm^2(from 0.661 g/cm^2 to 0.683 g/cm^2).<br><br>**Clinical efficacy:**<br>Cured (24)	(Xiao, 2016)

supplement with vitamin D chewable tablets (700 mg/day).<br>**TCM (35 cases)**: Yigu Soup<br>**Treatment duration:** not known<br>**Measurements:** BMD; serum RANKL and osteoprotegerin (OPG)<br>**Criteria for clinical efficacy:** Same as above **Criteria ③**	Significant effect (7)<br>Effective (3)<br>Ineffective (1)<br>Overall efficacy: 97.1%<br>Control treatment: 77.1%	
**Prescription [EE]:** Gengnian Gushu Prescription<br>**Percentage of *SM*:** 6.98%<br>**Types of clinical trials:** Single center RCT<br>**Patients**: 57 cases of osteoporosis<br>**Control (28 cases)**: Calcitriol (0.5 μg/day) for 2 months.<br>**TCM (29 cases)**: Gengnian Gushu Prescription for 2 months.<br>**Measurements:** VAS pain score, serum Ca, P, ALP<br>**Criteria for clinical efficacy:** Clinical control: disappearance or nearly disappearance of clinical symptoms, N > 95%; Significant effect: significant improvement of symptoms, N between 60%~95%; Effective: improvement of symptoms, N between 30%~60%; Ineffective: symptoms remained the same or even aggravated, N < 30%.	Significant decrease in VAS pain scores and serum levels of ALP;<br>Significant increase in serum levels of Ca and P.<br>**Clinical efficacy:**<br>Clinical control (5)<br>Significant effect (13)<br>Effective (8)<br>Ineffective (3)<br>Overall efficacy: 89.7%<br>Control treatment: 71.4%	(Wang, 2020)
**Prescription [FF]:** XianLing GuBao Capsule<br>**Prescription [GG]:** GuShuKang Capsule<br>**Percentage of *SM*:** Not known<br>**Types of clinical trials:** Single center RCT<br>**Patients:** 140 cases of osteoporosis<br>**Control (48 cases)**: Alendronate sodium for 6 months.<br>**TCM 1 (47 cases)**: XianLing GuBao Capsule for 6 months.<br>**TCM 2 (45 cases)**: GuShuKang Capsule for 6 months.<br>**Measurements:** BMD; serum Ca and ALP	**TCM 1:** BMD T-score significantly increased by 4.12 g/cm^2 (from -3.47 g/cm^2 to 0.65 g/cm^2);<br>**TCM 2:** BMD T-score significantly increased by 2.52 g/cm^2 (from -3.54 g/cm^2 to -1.02 g/cm^2);<br>Significant decrease in serum levels of ALP;<br>Significant increase in serum levels of Ca;<br>XianLing GuBao Capsule is better than GuShuKang Capsule in preventing osteoporosis.	(Shi et al., 2020)

Chinese prescription Pinyin Name and constituents:

[A] *Astragalus membranaceus* (Fisch.) Bge. var. *mongholicus* (Bge.) Hsiao (Astragali Radix) 15g, Epimedii Folium 12g, *Eclipta prostrata* L. (Ecliptae Herba) 12g, **Salviae Miltiorrhizae Radix et Rhizoma** 9g, *Sinomenium acutum* (Thunb.) Rehd. et Wils. (Sinomenii Caulis) 9g, *Achyranthes bidentata* Bl. (Achyranthis Bidentatae Radix) 9g.

[B] Epimedii Folium 15g, *Drynaria fortunei* (Kunze) J.Sm. (Drynariae Rhizoma) 15g, *Psoralea corylifolia* L. (Psoralleae Fructus) 15g, *Eucommia ulmoides* Oliv. (Eucommiae Cortex)10g, *Dipsacus asper* Wall. ex Henry (Dipsaci Radix) 10g, *Cornus officinalis* Sieb.et Zucc. (Corni Fructus) 12g, (Rehmanniae Radix Praeparata) 12g, *Pueraria lobata* (Willd.) Ohwi (Puerariae Lobatae Radix) 10g, **Salviae Miltiorrhizae Radix et Rhizoma** 10g, Angelicae Sinensis Radix 10g, *Spatholobus suberectus* Dunn (Spatholobi Caulis) 10g, *Glycyrrhiza uralensis* Fisch. (Glycyrrhizae Radix et Rhizoma) 5g, et al.

[C] Epimedii Folium 6g, **Salviae Miltiorrhizae Radix et Rhizoma** 3g, Rehmanniae Radix Praeparata 3g, Astragali Radix 1g, Drynariae Rhizoma 1g, Eucommiae Cortex 1g.

[D] Rehmanniae Radix Praeparata 20 g, **Salviae Miltiorrhizae Radix et Rhizoma** 15g, *Dioscorea opposita* Thunb. (Dioscoreae Rhizoma) 10g, Epimedii Folium 10g, *Chinemys reevesii* (Gray) (Testudinis Carapacis et Plastri Colla) 10g, *Cervus elaphus* Linnaeus (Cervi Cornus Colla) 10g, *Cistanche deserticola* Y.C.Ma (Cistanches Herba) 10g, Achyranthis Bidentatae Radix 10g, *Cuscuta australis* R. Br. (Cuscutae Semen)10g, Corni Fructus 10g, *Lycium barbarum* L. (Lycii Fructus) 8g, *Panax notoginseng* (Burk) F.H.Chen (Notoginseng Radix et Rhizoma) 3g.

[E] Angelicae Sinensis Radix, Astragali Radix, *Rehmannia glutinosa* Libosch. (Rehmanniae Radix Praeparata), *Ligusticum*

chuanxiong Hort. (Chuanxiong Rhizoma), Notoginseng Radix et Rhizoma, Achyranthis Bidentatae Radix, Corni Fructus, *Poria cocos* (Schw.) Wolf (Poria), Manis pentadactyla, **Salviae Miltiorrhizae Radix et Rhizoma**, Macaca mulatta Zimmermann, et al.

[F] Achyranthis Bidentatae Radix 10g, **Salviae Miltiorrhizae Radix et Rhizoma**10g, Eucommiae Cortex 10g, Psoralleae Fructus 10g, Epimedii Folium 10g, *Carthamus tinctorius* L. (Carthami Flos) 10g, Dioscoreae Rhizoma10g, *Curculigo orchioides* Gaertn. (Curculiginis Rhizoma) 10g, Corni Fructus 10g, Lycii Fructus 10g, Rehmanniae Radix Praeparata 15g.

[G] Rehmanniae Radix Praeparata10g, Psoralleae Fructus 15g, Eucommiae Cortex15g, Dioscoreae Rhizoma 30g, *Buthus martensii* Karsch (Scorpio) 10g, Achyranthis Bidentatae Radix 10g, **Salviae Miltiorrhizae Radix et Rhizoma** 10g, Angelicae Sinensis Radix 10g, *Angelica pubescens* Maxim. f. *biserrata* Shan et Yuan (Angelicae Pubescentis Radix) 10g, *Notopterygium incisum* Ting ex H. T. Chang (Notopterygii Rhizoma et Radix) 10g, Chuanxiong Rhizoma 30g.

[H] Rehmanniae Radix Praeparata 20g, Corni Fructus 10g, Eucommiae Cortex 15g, Dioscoreae Rhizoma10g, Drynariae Rhizoma10g, **Salviae Miltiorrhizae Radix et Rhizoma** 15g, Achyranthis Bidentatae Radix 10g, Epimedii Folium 10g, Lycii Fructus 15g, *Cyperus rotundus* L. (Cyperi Rhizoma) 10g, *Paneonia suffruticosa* Andr. (Moutan Cortex)15g, Poria10g, Angelicae Sinensis Radix 10g.

[I] Rehmanniae Radix Praeparata 10g, Eucommiae Cortex 10g, Corni Fructus 10g, Cervi Cornus Colla 3g, Drynariae Rhizoma 10g, Achyranthis Bidentatae Radix 10g, Astragali Radix 20g, Glycyrrhizae Radix et Rhizoma 3g, Notoginseng Radix et Rhizoma 5g, **Salviae Miltiorrhizae Radix et Rhizoma** 9g, Chuanxiong Rhizoma 10g.

[J] Astragali Radix 15g, Epimedii Folium 12g, Ecliptae Herba 12g, **Salviae Miltiorrhizae Radix et Rhizoma** 9g, Sinomenii Caulis 9g, Achyranthis Bidentatae Radix 9g.

[K] Epimedii Folium 10g, Curculiginis Rhizoma 15g, Angelicae Sinensis Radix 30g, *Agrimonia pilosa* Ledeb. (Agrimoniae Herba) 30g, *Cynomorium songaricum* Rupr. (Cynomorii Herba) 10g, Cuscutae Semen 15g, Astragali Radix 15g, **Salviae Miltiorrhizae Radix et Rhizoma** 20g, Spatholobi Caulis 15g, *Anemarrhena asphodeloides* Bge. (Anemarrhenae Rhizoma) 10g, *Phellodendron chinense* Schneid. (Phellodendri Chinensis Cortex) 10g, Poria 15g, *Ostrea gigas* Thunberg (Ostreae Concha) 30g, Achyranthis Bidentatae Radix 15g.

[L] Eucommiae Cortex, Drynariae Rhizoma, *Cervus elaphus Linnaeus* (Cervi Cornu), Chinemys reevesii (Gray) (Testudinis Carapax et Plastrum), Dipsaci Radix, **Salviae Miltiorrhizae Radix et Rhizoma**, Carthami Flos, Poria, et al.

[M] Rehmanniae Radix Praeparata 30g, Dioscoreae Rhizoma 20g, Corni Fructus 20g, Eucommiae Cortex 15g, Achyranthis Bidentatae Radix 20g, Drynariae Rhizoma 20g, Psoralleae Fructus 20g, *Ligustrum lucidum* Ait. (Ligustri Lucidi Fructus) 20g, Ecliptae Herba 20g, **Salviae Miltiorrhizae Radix et Rhizoma**20g, Angelicae Sinensis Radix 20g, Anemarrhenae Rhizoma 15g, Glycyrrhizae Radix et Rhizoma10g.

[N] Epimedii Folium 30g, Astragali Radix 20g, Eucommiae Cortex 15g, Dioscoreae Rhizoma15g, **Salviae Miltiorrhizae Radix et Rhizoma** 15g, Notoginseng Radix et Rhizoma 10g.

[O] *Gentiana macrophylla* Pall. (Gentianae Macrophyllae Radix) 9g, Chuanxiong Rhizoma 6g, *Prunus persica* (L.) Batsch (Persicae Semen) 9g, Carthami Flos 9g, Glycyrrhizae Radix et Rhizoma 6g, Notopterygii Rhizoma et Radix 6g, *Commiphora myrrha* Engl. (Myrrha) 6g, Angelicae Sinensis Radix 9g, *Trogopterus xanthipes* (Faeces Trogopterori) 6g, Cyperi Rhizoma 6g, Achyranthis Bidentatae Radix 9g, **Salviae Miltiorrhizae Radix et Rhizoma** 6g, Notoginseng Radix et Rhizoma 6g, *Pheretima aspergillum* (E.Perrier) (Pheretima) 6g.

[P] *Trionyx sinensis* Wiegmann (Trionycis Carapax) 30g, Os Draconis 30g, Testudinis Carapax et Plastrum 20g, Ostreae Concha 20g, Angelicae Sinensis Radix 25g, *Polygonum multiflorum* Thunb. (Polygoni Multiflori Radix) 30g, **Salviae**

Miltiorrhizae Radix et Rhizoma 15g, Achyranthis Bidentatae Radix 30g, Poria 50g, *Dioscorea spongiosa* J.Q.Xi, M. Mizuno et W. L. Zhao (Dioscoreae Spongiosae Rhizoma) 20g, Polygoni Multiflori Radix 15g, Astragali Radix 15g.

[Q] Rehmanniae Radix Praeparata, Epimedii Folium, Dioscoreae Rhizoma, Lycii Fructus, **Salviae Miltiorrhizae Radix et Rhizoma**, Cervi Cornus Colla, Eucommiae Cortex, Achyranthis Bidentatae Radix, Glycyrrhizae Radix et Rhizoma, et al.

[R] Rehmanniae Radix Praeparata 30g, Corni Fructus 12g, Epimedii Folium 12g, Eucommiae Cortex 12g, Cuscutae Semen 12g, Spatholobi Caulis 30g, **Salviae Miltiorrhizae Radix et Rhizoma** 30g, Dipsaci Radix 15g, Cervi Cornus Colla 15g, Drynariae Rhizoma 15g, Dioscoreae Rhizoma 15g, Angelicae Sinensis Radix 15g, Achyranthis Bidentatae Radix 15g, Chuanxiong Rhizoma 15g, Angelicae Pubescentis Radix 10g, Whitmania pigra Whitman (Hirudo) 10g, *Alisma orientale* (Sam.) Juzep. (Alismatis Rhizoma) 10g, Glycyrrhizae Radix et Rhizoma 6g.

[S] **Salviae Miltiorrhizae Radix et Rhizoma** 20g, Rehmanniae Radix Praeparata 20g, Astragali Radix 20g, *Atractylodes macrocephala* Koidz. (Atractylodis Macrocephalae Rhizoma) 20g, Drynariae Rhizoma 15g, Dioscoreae Rhizoma20g, Eucommiae Cortex 15g, Epimedii Folium 15 g, Poria20g, *Cibotium barometz* (L.) J. Sm. (Cibotii Rhizoma) 15g, Cyperi Rhizoma 15g, *Acanthopanax senticosus* (Rupr. et Maxim.) Harms (Acanthopanacis Senticosi Radix et Rhizoma Seu Caulis) 15g, *Saposhnikovia divaricata* (Turcz.) Schischk. (Saposhnikoviae Radix) 9g, Ligustri Lucidi Fructus 15g, *Eupolyphaga sinensis* Walker (Eupolyphaga Steleophaga) 10g, Notoginseng Radix et Rhizoma 10g, Pheretima10g, Glycyrrhizae Radix et Rhizoma 6g.

[T] Astragali Radix 30g, **Salviae Miltiorrhizae Radix et Rhizoma** 20g, Poria 20g, Atractylodis Macrocephalae Rhizoma 20g, Dioscoreae Rhizoma 20g, Epimedii Folium 15g, Drynariae Rhizoma 15g, Cibotii Rhizoma 15g, Eucommiae Cortex 15g, Ligustri Lucidi Fructus 15g, Acanthopanacis Senticosi Radix et Rhizoma Seu Caulis 15g, Cyperi Rhizoma 15g, Notoginseng Radix et Rhizoma 10g, Eupolyphaga Steleophaga10g, Pheretima 10g, Saposhnikoviae Radix 9g, Glycyrrhizae Radix et Rhizoma 6g.

[U] Astragali Radix 30g, **Salviae Miltiorrhizae Radix et Rhizoma**18g, Chuanxiong Rhizoma 6g, Psoralleae Fructus 12g, Drynariae Rhizoma15g, Dipsaci Radix 9g, Dioscoreae Rhizoma 30g, Glycyrrhizae Radix et Rhizoma 6g.

[V] *Codonopsis pilosula* (Franch.) Nannf. (Codonopsis Radix) 75g, **Salviae Miltiorrhizae Radix et Rhizoma**150g, Epimedii Folium 30g, Curculiginis Rhizoma 150g, Psoralleae Fructus 50g, Rehmanniae Radix Praeparata 75g, *Morinda officinalis* How (Morindae Officinalis Radix) 50g, Eucommiae Cortex 50g.

[W] Rehmanniae Radix Praeparata 15g, Dioscoreae Rhizoma 10g, Corni Fructus 10g, Alismatis Rhizoma 10g, Anemarrhenae Rhizoma 10g, **Salviae Miltiorrhizae Radix et Rhizoma** 15g, Achyranthis Bidentatae Radix 10g, Cuscutae Semen10g, Epimedii Folium 10g, Psoralleae Fructus 10g, Astragali Radix 30g, Glycyrrhizae Radix ET Rhizoma 6g.

[X] Epimedii Folium 12g, Cervi Cornus Colla 15g, *Polygonatum kingianum* Coll.et Hemsl. (Polygonati Rhizoma) 12g, *Astragalus complanatus* R. Br. (Astragali Complanati Semen) 15g, Polygoni Multiflori Radix 15g, Astragali Radix 30g, Dioscoreae Rhizoma 30g, Puerariae Lobatae Radix 30g, **Salviae Miltiorrhizae Radix et Rhizoma** 30g, *Rheum palmatum* L. (Rhei Radix et Rhizoma) 10g.

[Y] Epimedii Folium, Rehmanniae Radix Praeparata, Astragali Radix, **Salviae Miltiorrhizae Radix et Rhizoma**, Drynariae Rhizoma et al.

[Z] Astragali Radix 30g, Angelicae Sinensis Radix 12g, Spatholobi Caulis 15g, Hirudo 3g, Drynariae Rhizoma 15g, Dioscoreae Rhizoma 30g, Chuanxiong Rhizoma 9g, *Lonicera japonica* Thunb. (Lonicerae Japonicae Flos) 15g, Achyranthis Bidentatae Radix 9g, **Salviae Miltiorrhizae Radix et Rhizoma** 12g, *Corydalis yanhusuo* W.T. Wang (Corydalis Rhizoma) 10g, Glycyrrhizae Radix ET Rhizoma 6g.

[AA] Astragali Radix 30g, *Rehmannia glutinosa* Libosch. (Rehmanniae Radix) 30g, Corni Fructus 9g, Lycii Fructus 9g, Ligustri Lucidi Fructus 9g, Cuscutae Semen 9g, Drynariae Rhizoma 15g, Epimedii Folium 9g, Ostreae Concha 30g, Angelicae Sinensis Radix 15g, Paeonia lactiflora Pall. (Paeoniae Radix Rubra) 15g, **Salviae Miltiorrhizae Radix et Rhizoma** 15g, Moutan Cortex 9g, Glycyrrhizae Radix ET Rhizoma 3g.

[BB] Epimedii Folium 12g, Achyranthis Bidentatae Radix 12g, Psoralleae Fructus 10g, Rehmanniae Radix Praeparata 12g, **Salviae Miltiorrhizae Radix et Rhizoma** 15g, Astragali Radix 20g, Poria 20g, Ostreae Concha 20g, *Taxillus chinensis* (DC.) Danser (Taxilli Herba) 12g, Angelicae Sinensis Radix12g, et al.

[CC] Rehmanniae Radix Praeparata 24g, Dioscoreae Rhizoma 12g, Lycii Fructus 12g, Corni Fructus 12g, Achyranthis Bidentatae Radix 9g, Cervi Cornus Colla 12g, Testudinis Carapacis et Plastri Colla 12g, Cuscutae Semen 12g, Angelicae Sinensis Radix 15g, **Salviae Miltiorrhizae Radix et Rhizoma** 15g, *Boswellia carterii* Birdw. (Olibanum) 15g, Myrrha 15g.

[DD] **Salviae Miltiorrhizae Radix et Rhizoma** 30g, Drynariae Rhizoma15g, Dioscoreae Rhizoma15g, Psoralleae Fructus 10g, Epimedii Folium 15g, Rehmanniae Radix15g.

[EE] Curculiginis Rhizoma 10g, Epimedii Folium 10g, Morindae officinalis radix 10g, Angelicae Sinensis Radix 10g, Phellodendri Chinensis Cortex 10g, Anemarrhenae Rhizoma 10g, Rehmanniae Radix Praeparata 15g, Dioscoreae Rhizoma 15g, Lycii Fructus 10g, Corni Fructus 15g, Achyranthis Bidentatae Radix 15g, Cuscutae Semen 10g, Dipsaci Radix 15g, Psoralleae Fructus 15g, **Salviae Miltiorrhizae Radix et Rhizoma** 15g, Drynariae Rhizoma 15g, Testudinis Carapax et Plastrum 15g.

[FF] Epimedii Folium, Rehmanniae Radix Praeparata, Drynariae Rhizoma, Astragali Radix, **Salviae Miltiorrhizae Radix et Rhizoma**, *Auricularia auricula* (L.ex Hook.)Underwood, *Cucumis satiuus* L.

[GG] Epimedii Folium, Dipsaci Radix, **Salviae Miltiorrhizae Radix et Rhizoma**, Anemarrhenae Rhizoma, Psoralleae Fructus, Rehmanniae Radix.

Reference

Bao, J. (2022). Clinical efficacy observation of Xianshen Bugu Yin for treating primary osteoporosis with kidney deficiency and blood stasis. [dissertation/master's thesis]. [Nanjing]: Nanjing University of Chinese Medicine.

Cai, Y., and Deng, D. (2015). Clinical observation of “Yiqi Huoxue Zhuanggu Formula”on bone mineral density and bone metabolism of elder patients with osteoporosis complicated with stable coronary heart disease. Shanghai J TCM. 49, 43–45. doi:10.16305/j.1007-1334.2015.03.031

Cao, J., Liang, J., Feng, J., Wang, Y., and Huang, M. (2021). Clinical study on Yishen Qianggu Mixture in treatment of senile osteoporosis with kidney-yang deficiency. J Hubei Univ TCM. 23, 88–90.

Chai, Y., Li, Q., Liu, F., Wen, Z., and Yang, J. (2015). Observation on the clinical effect of Bushen Huoxue prescription in the treatment of senile female osteoporosis. Chin J Clin. Ration. Drug Use. 8, 83–84. doi:10.15887/j.cnki.13-1389/r.2015.01.048

Chen, H. (2020). Effects of gujian decoction on estrogen, balance and lumbar function in postmenopausal osteoporosis with kidney deficiency and blood stasis. [dissertation/master's thesis]. [Fujian]: Fujian University of Traditional Chinese Medicine.

Bao, J. (2022). Clinical efficacy observation of Xianshen Bugu Yin for treating primary osteoporosis with kidney deficiency and blood stasis. [dissertation/master's thesis]. [Nanjing]: Nanjing

University of Chinese Medicine
Cai, Y., and Deng, D. (2015). Clinical observation of “Yiqi Huoxue Zhuanggu Formula”on bone mineral density and bone metabolism of elder patients with osteoporosis complicated with stable coronary heart disease. ShangHai J TCM. 49, 43–45. doi:10.16305/j.1007-1334.2015.03.031
Cao, J., Liang, J., Feng, J., Wang, Y., and Huang, M. (2021). Clinical study on Yishen Qianggu Mixture in treatment of senile osteoporosis with kidney-yang deficiency. J HuBei Univ TCM. 23, 88–90. DOI is not available.
Chai, Y., Li, Q., Liu, F., Wen, Z., and Yang, J. (2015). Observation on the clinical effect of Bushen Huoxue prescription in the treatment of senile female osteoporosis. Chin J Clin. Ration. Drug Use. 8, 83–84. doi:10.15887/j.cnki.13-1389/r.2015.01.048
Chen, H. (2018). Effects of gujian decoction on estrogen, balance and lumbar function in postmenopausal osteoporosis with kidney deficiency and blood stasis. [dissertation/master's thesis]. [Fujian]: Fujian University of Traditional Chinese Medicine
Chen, H.,Wang, A., Xiao, Z., Zhu, J., Wang, F., Xu, Z., et al. (2020). Curative effect of acupuncture combined with tanshinone Ⅱ A sulfonate injection on primary osteoporosis. J Inn. Mong. TCM. 39, 124–126. doi:10.16040/j.cnki.cn15-1101.2020.07.075
Chen, L., Ding, H., Liao, S., Yu, D., andWei, B. (2017b). Observation on the curative effect of comprehensive treatment for primary osteoporosis. J Pract. TCM. 33(08), 922. DOI is not available.
Chu, M. (2019). Impacts on lumbar vertebrae biomechanics and the TCM symptoms of primary osteoporosis of kidney deficiency and blood stasis pattern treat with gujian decoction. [dissertation/master's thesis]. [Fujian]: Fujian University of Traditional Chinese Medicine
Dai, B. (2022). Observation on clinical effect of Bushen Huoxue Decoction on kidney deficiency and blood stasis syndrome of osteoporosis. [dissertation/master's thesis]. [Hunan]: Hunan University of Chinese Medicine
Dai, J. (2020). A double-blind multicenter randomized placebo-controlled trial Intervention of Osteopenia with low back pain by JianYaoMiGu Granule. [dissertation/master's thesis]. [Shanghai]: Shanghai University of Traditional Chinese Medicine
Fu, R. (2021). Clinical effect of Bushen Huoxue prescription on type 2 diabetic osteoporosis. [dissertation/master's thesis]. [Guangzhou]: Guangzhou University of Chinese Medicine
Gong, X. (2019). The clinical observation of Zuogui Pill combined with Huoluo Xiaolingdan in treating osteoporosis (kidney deficiency and blood stasis syndrome). [dissertation/master's thesis]. [Hunan]: Hunan University of Chinese Medicine
Han, B. (2016). Observation of curative effect of Bushen Huoxue Decoction combined with caltrate to treat postmenopausal primary osteoporosis. Mod. J Integr. TCM West. Med. 25, 2207–2209. doi: 10.3969/j.issn.1008-8849.2016.20.014
He, J. (2019). Comparative study on the effect of jianyao migu prescription combined with calcitriol in improving postmenopausal osteoporosis in the community. [dissertation/master's thesis]. [Shanghai]: Shanghai University of Traditional Chinese Medicine
Hong, F. (2022). Precursive Blood-repellent bruises for clinical study of postmenopausal osteoporosis. [dissertation/master's thesis]. [ZheJiang]: Zhejiang Chinese Medical University
Jiang, Z. (2015). Clinical effect of Bushen Huoxue prescription on osteoporosis in elderly women. [dissertation/master's thesis]. [Hebei]: Hebei Medical University
Kong, G. (2014). The clinical research on treatment of osteoporosis treasure of kidney and spleen deficiency of two perimenopausal osteoporosis. [dissertation/master's thesis]. [Shandong]: Shandong University of Traditional Chinese Medicine
Li, G. (2018). Clinical research of Guben Huoxue Prescription in the treatment of diabetic osteoporosis.

[dissertation/master's thesis]. [Shandong]: Shandong University of Traditional Chinese Medicine

Li, Z. (2021). Clinical efficacy observation of YiYin ZhuangGu Pill in the treatment of postmenopausal osteoporosis of liver-kidney deficiency type. [dissertation/master's thesis]. [Heilongjiang]: Heilongjiang University of Chinese Medicine

Liu, C., Yang, G., and Ding, H. (2015). Clinical study of Bielong granules in the treatment of perimenopausal osteoporosis. J Chin Prescr. drug. 13, 43–44. doi: 10.3969/j.issn.1671-945X.2015.03.032

Mei, Y. (2014). Invigorating the spleen method of clinical research for the treatment of primary osteoporosis. [dissertation/master's thesis]. [Hubei]: Hubei University of Chinese Medicine

Pan, Z. (2014). Clinical studies of osteoporosis Young Bushenhuoxue treated. [dissertation/master's thesis]. [Chengdu]: Chengdu University of TCM

Ru, G. (2016). Study on Bushen Jiangu Decoction combined with alenophosphate sodium tablets on 38 cases of osteoporosis of urban white-collar workers. TCM Res. 29, 25–27. doi: 10.3969/j.issn.1001-6910.2016.11.11

Shi, M. (2018). Clinical observation of Yishenjiangu Decoction on treatment of early type 2 diabetic nephropathy complicated with osteoporosis syndrome of kidney deficiency and blood stasis. [dissertation/master's thesis]. [Nanjing]: Nanjing University of Chinese Medicine

Shi, X., Feng, L., Li, H., Lu, C., and Zhang, X. (2020). Effects of different Chinese medicine preparations on blood calcium, blood alkaline phosphatase and bone mineral density in osteoporosis patients. Guangming J TCM. 35, 3346–3348. doi: 10.3969/j.issn.1003-8914.2020.21.012

Shuai, L. (2018) Clinical study of GuShuKang Capsule in the treatment of type 2 dibetic mellitus. [dissertation/master's thesis]. [Shanxi]: Shanxi University of Chinese Medicin

Su, G., Yang, H., and Feng, Z. (2020). Curative effect of using self-made danshen huangqi decoction combined with calcitonin in the treatment of elderly with osteoporosis and its influences on bone mineral density and bone metabolism. J Sichuan TCM. 38, 149–152. DOI is not avaliable

Wang, C., Zhu, W., Lin, H., and Lu, G. (2022). Clinical effect observation on selfdesigned Yiqi Bushen Huoxue decoction in the treatment of primary osteoporosis. Clin. J TCM. 14, 62–64. doi: 10.3969/j.issn.1674-7860.2022.18.019

Wang, H. (2022). Danshen Dihuang Zhitong prescription combined with conventional western medicine treatment Clinical study of osteoporosis in senile patients. Henan TCM. 42, 1245–1248. doi: 10.16367/j.issn.1003-5028.2022.08.0266

Wang, Y. (2018). Clinical Study of YiQi TongLuo Prescription on the treatment elderly type 2 diabetes combine with osteoporosis. [dissertation/master's thesis]. [Shandong]: Shandong University of Traditional Chinese Medicine

Wang, Z. (2020). The clinical observation of Gennian Gushu Decoction in the treatment of liver and kidney Yin deficiency type osteoporosis. [dissertation/master's thesis]. [Hunan]: Hunan University of Chinese Medicine

Xiao, Z. (2016). The effect of Yigu Decoction on bone density, serum OPG and RANKL of osteoporosis patients combine with deficiency of kidney, Qi and blood. Med Forum. 20(29), 4128–4129. doi: 10.19435/j.1672-1721.2016.29.064

Xie, J. (2018). Evaluation of zhuanggufang decoction powder’s curative effect on spleen and kidney merged stagnation blood type of postmenopausal low bone mass population. [dissertation/master's thesis]. [Guangxi]: Guangxi University of Chinese Medicine

Xu, W. (2020). Clinical obervation on the treatment of diabetic osteoporosis by strengthening spleen, tonifying dikney and activating blood circulation and its effect on PINP and β-CTX. [dissertation/master's thesis]. [Anhui]: Anhui University of Chinese Medicine

Yang, H., Wang, Y., and Zhang, H. (2017). Clinical study of 40 cases of primary osteoporosis treated by combination of acupuncture and medicine. J Jiangsu TCM. 49, 59–60. doi: 10.3969/j.issn.1672-397X.2017.09.025

Yang, Z. (2015). The clinical research on the effect of Bushen Jiangu Decoction in the treatment of primary osteoporosis. [dissertation/master's thesis]. [Nanjing]: Nanjing University of Chinese Medicine

Zhang, F. (2019). Clinical observation on the therapeutic effect of Jiawei dioxins decoction on postmenopausal osteoporosis with renal deficiency and blood stasis. [dissertation/master's thesis]. [Gansu]: Gansu University of Chinese Medicine

Zheng, X. (2018). Effect of GuJianFang on bone metabolism indexes of primary osteoporosis with kidney deficiency and blood stasis. [dissertation/master's thesis]. [Fujian]: Fujian University of Traditional Chinese Medicine
